# Ultra-Accurate Correlation between Precursor and Fragment Ions in Two-Dimensional Mass Spectrometry: Acetylated vs Trimethylated Histone Peptides

**DOI:** 10.1021/jasms.2c00319

**Published:** 2023-03-17

**Authors:** Michael Palasser, Sarah V. Heel, Marc-André Delsuc, Kathrin Breuker, Maria A. van Agthoven

**Affiliations:** † Institute for Organic Chemistry, University of Innsbruck, 80/82 Innrain, 6020 Innsbruck, Austria; ‡ Institut de Génétique et de Biologie Moléculaire et Cellulaire, INSERM U596, UMR 7104, Université de Strasbourg, 1 rue Laurent Fries, 67404 Illkirch-Graffenstaden, France; § CASC4DE, Pôle API, 300 Bd. Sébastien Grant, 67400 Illkirch-Graffenstaden, France

## Abstract

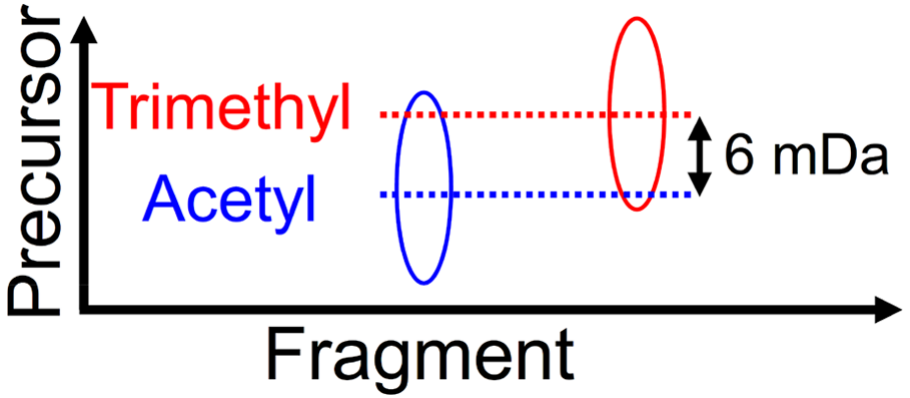

Two-dimensional mass spectrometry (2D MS) is a method for tandem mass spectrometry in which precursor and fragment ions are correlated by manipulating ion radii rather than by ion isolation. A 2D mass spectrum contains the fragmentation patterns of all analytes in a sample, acquired in parallel. We report ultrahigh-resolution narrowband 2D mass spectra of a mixture of two histone peptides with the same sequence, one of which carries an acetylation and the other a trimethylation (*m*/*z* 0.006 difference). We reduced the distance between data points in the precursor ion dimension and compared the accuracy of the precursor-fragment correlation with the resolving power. We manage to perform label-free quantification on the histone peptide mixture and show that precursor and fragment ions can be accurately correlated even though the precursor ions are not resolved. Finally, we show that increasing the resolution of a 2D mass spectrum in the precursor ion dimension too far can lead to a decline in the signal-to-noise ratio.

## Introduction

In tandem mass spectrometry, ions undergo fragmentation to yield structural information beyond the measurement of their mass-to-charge (*m*/*z*) ratio. For complex samples, one of the most important issues is the correlation between fragment ions and their precursors. In most standard applications, this correlation is achieved by isolating a single ion species before fragmentation.^1^ With highly complex samples, ion isolation needs to be performed with narrow *m*/*z* windows, which can be challenging. To date, stored-waveform inverse Fourier transform and correlated harmonic excitation fields isolation are the two methods that offer the narrowest *m*/*z* windows in ion isolation.^2−5^

Two-dimensional mass spectrometry (2D MS) correlates precursor and fragment ions by manipulating their trajectories, typically in a quadrupolar ion trap or in an ion cyclotron resonance (ICR) cell.^6−11^ Other methods such as two-dimensional partial covariance mass spectrometry (2D PC MS) correlate fragment ions with one another.^12−14^ Because 2D MS does not require ion isolation and relies on signal multiplexing, it can measure the structural information on analytes in complex samples in a parallel instead of in a serial way.^6^

The pulse sequence for 2D FT-ICR MS (two-dimensional Fourier transform ion cyclotron resonance mass spectrometry) was developed by Pfändler et al. and has been optimized for various fragmentation methods, such as infrared multiphoton dissociation, electron capture dissociation (ECD), infrared-activated ECD, electron-induced dissociation, and ultraviolet dissociation.^15−21^ Programs have been developed for data processing and analysis, and algorithms have been developed for noise reduction.^6,22−24^ Applications for 2D MS include small molecules, proteomics (bottom-up and top-down), polymer analysis, and agrochemicals.^20,21,25−32^ A narrowband method has been developed for high resolution precursor-fragment correlation over small *m*/*z* ranges as well as strategies for phase correction to improve both the resolving power and the signal-to-noise ratio of 2D mass spectra.^33−35^

Recent studies by Marzullo *et al.* and by Paris *et al.* show that precursor and fragment ions can be correlated accurately, even for multiple precursor ions peaks that are not properly resolved in the vertical precursor ion dimension.^21,31^ In this study, we performed narrowband 2D MS on an FT-ICR mass spectrometer, in phase-corrected absorption mode, on the mixture of trimethylated and acetylated forms of histone peptide with an *m*/*z* difference of 0.006. We varied the frequency range in the precursor ion dimension and compared the resolving power, signal-to-noise, and precursor-fragment correlation to probe the limits of performance of 2D MS in terms of accurate precursor-fragment correlation.

Here, we define resolution of a spectrum as the distance between two adjacent data points, resolution of a peak as its full-width at half-maximum (FWHM), resolving power as the measurement of a peak centroid divided by the FWHM of the peak, and precursor-fragment correlation as the capacity to accurately assign both a precursor and a fragment ion for a peak in a 2D mass spectrum.

## Experimental Methods

### Sample Preparation

Milli-Q water (Merck Millipore, Darmstadt, Germany), methanol, and acetic acid (VWR, Vienna, Austria) were used in all experiments. C-terminal GK-biotinylated histone H3 sequences (amino acid residues 21–44) with native modifications (acetylation at K27 and trimethylation at K36) were purchased (AnaSpec, Fremont, CA, USA) with a purity of >95%, in which K27 of the full-length histone corresponds to K7 of the model peptides and K36 in the histone corresponds to K16 in the peptides.

Peptides were desalted using MWCO 2000 Vivaspin centrifugal concentrators (Sartorius, Göttingen, Germany) at 7900 rcf, 6× ammonium acetate 100 mM (Sigma, Vienna, Austria), and 6× H_2_O. Peptide concentration was determined by UV absorption at 280 nm using an Implen Nano PhotometerTM (Implen, München, Germany). For electrospray ionization, equimolar mixtures (0.2 μM each) of acetylated and trimethylated peptides (referred to as “K7 Ac” and “K16 3m”, respectively, in the text) in 50:50 H_2_O/CH_3_OH and 1% vol CH_3_COOH, pH ∼ 3.0 were prepared from 100 μM stock solutions of each peptide in H_2_O.

### Instrument Parameters

All mass spectra were acquired on a 7 T Apex Ultra FT-ICR mass spectrometer (Bruker Daltonik, GmbH, Bremen, Germany) with an electrospray ion source operated in positive mode and direct injection at a flow rate of 70 μL/h.^36^ Ions were transferred to the Infinity ICR cell through a series of focusing lenses.^37^

The mass spectrum of the histone peptide mixture was recorded over 20 scans with a 2 M data point transient (2.93 s) after ion accumulation of 0.1 s in the first hexapole and transfer to the second hexapole. The mass range was *m*/*z* 303.3–1500 (corresponding to a frequency range of 357.143–72.171 kHz). The excitation sweep had an amplitude of 80 V_pp_ with 20 μs/frequency (each frequency step is 624.64 Hz).

The four 2D mass spectra were acquired after ion accumulation of 0.2s in the first hexapole and transfer to the second hexapole. Ions were isolated in the quadrupole at *m*/*z* 494 with an isolation window of *m*/*z* 30. The fragmentation method was ECD with a hollow cathode, with the heater set at 1.2 A, the lens at 20 V, the bias at 2.0 V, and the irradiation at 20 ms.^38^

**Scheme 1:**
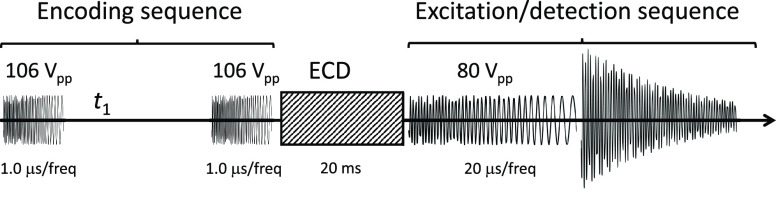
Pulse Sequence for 2D Mass Spectrometry

The pulse sequence for the 2D MS experiment is shown in Scheme 1. The two pulses in the encoding sequence were set at 106 V_pp_ amplitude with 1.0 μs/frequency, and the pulse in the excitation/detection sequence was set at 80 V_pp_ amplitude with a 20 μs/frequency (each frequency step is 624.64 Hz). These parameters were optimized with the same method that has been discussed in previous articles.^18,19^ The encoding delay *t*_1_ was incremented 1024 times in each experiment. Transients were acquired over 1 scan per value of *t*_1_ with 512 k data points (0.734 s). All pulses in the pulse sequence have the same frequency range. All four 2D mass spectra were acquired in narrowband mode, with different frequency ranges for the encoding and excitation pulses to provide for the desired frequency range in the final 2D mass spectra.^35^ All these parameters are shown in Table 1. For a detailed explanation of narrowband mode 2D MS, see Scheme S1 in the Supporting Information.

**Table 1:** Experimental Conditions of the 2D Mass Spectra

Nyquist frequency (kHz)	*t*_1_ increment (μs)	Frequency range of all pulses (kHz)	Horizontal *m*/*z* range	Number of foldovers	Vertical *m*/*z* range
10	50	357.143–74.660	303.3–1450	14	482.011–504.438
4	125	357.143–72.171	303.3–1500	36	491.828–500.916
2	250	357.143–73.895	303.3–1465	72	492.438–496.952
1	500	357.143–74.402	303.3–1455	144	493.539–495.796

Four MS/MS spectra were acquired for use in determining the coefficients of the horizontal quadratic phase correction function with the same frequency ranges as the 2D mass spectra listed in Table 1. The front-end conditions, ECD parameters, and transients of these MS/MS spectra were identical to the ones in the 2D mass spectrum. The MS/MS spectra were acquired with 500 accumulated scans each.

### Data Processing

Both the one-dimensional (1D) and 2D mass spectra were processed and visualized using the Spectrometry Processing Innovative Kernel (SPIKE) software (available at www.github.com/spike-project, accessed on June 1, 2021) developed by the University of Strasbourg (Strasbourg, France) and CASC4DE (Illkirch-Graffenstaden, France) in the 64-bit Python 3.7 programming language on an open-source platform distributed by the Python Software Foundation (Beaverton, OR, USA).^22^ Processed data files were saved using the HDF5 file format.

The 1D mass spectra were apodized with a square sine-bell window with a maximum at 0.15 and zero-filled to 4096 data points before Fourier transformation and quadratic phase-correction and baseline correction. The 2D mass spectra were digitally demodulated, apodized with a sine-bell window with a maximum at 0.15, zero-filled twice, denoised with the SANE algorithm (with a rank of 30), and phase-corrected quadratically along the horizontal axis and linearly along the vertical access, before the frequencies were offset to recalculate the original frequency range in the vertical dimension.^23,24,34,35^ The size of the resulting data sets was 1,048,576 data points horizontally (fragment ion dimension) by 2048 data points vertically (precursor ion dimension).

Peak-picking for fragment assignments and relative quantification was achieved by extracting horizontal fragment ion scans for each precursor isotope and baseline-correction before accumulation. Peak-picking was conducted on the resulting data set, followed by centroiding and frequency-to-mass quadratic internal conversion.^39^ To determine the correlation between precursor and fragment ions, we peak-picked by extracting the horizontal fragment ion scan of the M or M+1 isotope of the precursor ion. Then the vertical precursor ion scan at the peak was extracted, baseline-corrected, and peak-picked. Each peak was centroided (polynomial fit). The 1D profile data from the vertical precursor ion scans and the centroid were used to generate a Lorentzian fit. All programs used are listed in the Supporting Information.

## Results and Discussion

Scheme 1 shows the pulse sequence for 2D MS on an FT-ICR mass spectrometer.^15−17^ During the encoding sequence, precursor ion radii are modulated. The first pulse increases the radius of all precursor ions, after which they are left to rotate at their cyclotron frequency during the encoding delay (*t*_1_). The ions accrue phase, which brings them out of phase with the second pulse and leads to radius modulation according to the ions’ cyclotron frequency and *t*_1_.^40^ Because ECD fragmentation is radius dependent, the abundance of the fragment ions is modulated according to *t*_1_ and the cyclotron frequencies of their precursors.^41^ The excitation/detection sequence brings all ions to high radius to acquire the transient.^6^

The pulse sequence is repeated for regularly incremented values of *t*_1_. Each transient is Fourier-transformed. Then the signal for each frequency is Fourier-transformed according to *t*_1_. After frequency-to-mass conversion, the 2D mass spectrum shows the fragmentation patterns of all analytes in the sample. In each dimension, the spectrum resolution increases with the number of data points and with decreasing frequency ranges.^42^

**Figure 1:**
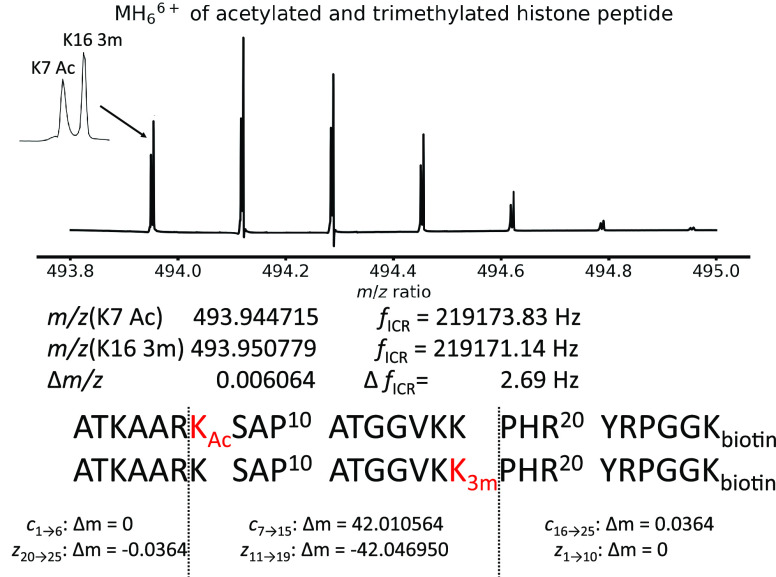
Sequence, *m*/*z* ratios, theoretical cyclotron frequencies, and isotope signals of the [M+6H]^6+^ ions of the K7 Ac and K16 3m histone peptides (full spectrum in Figure S1 in the Supporting Information).

Figure 1 shows the sequence (which corresponds to residues 21 to 44 of the histone H3 protein) and modifications of the two histone peptides used in this study. Both peptides are biotinylated at K26, in addition to which one is acetylated at lysine 7 (K7 Ac) and the other trimethylated at lysine 16 (K16 3m). The mass difference between the two histone peptides is 36 mDa.

Figure 1 shows the isotopic distribution of the [M+6H]^6+^ ions in the 1D mass spectrum of a ∼ 1:1 mixture of the two histone peptides (full spectrum in Figure S1 in the Supporting Information). The mass spectrum was acquired with 2 M data points and a Nyquist frequency of 357.143 kHz (the length of the transient was 2.936 s). Processing was achieved with two zero-fills in phase-corrected absorption mode. The peaks of the K7 Ac and K16 3m peptides (which are separated by *m*/*z* 0.006 or 2.7 Hz in cyclotron frequency at *z* = 6) are nearly baseline-resolved.

**Scheme 2:**
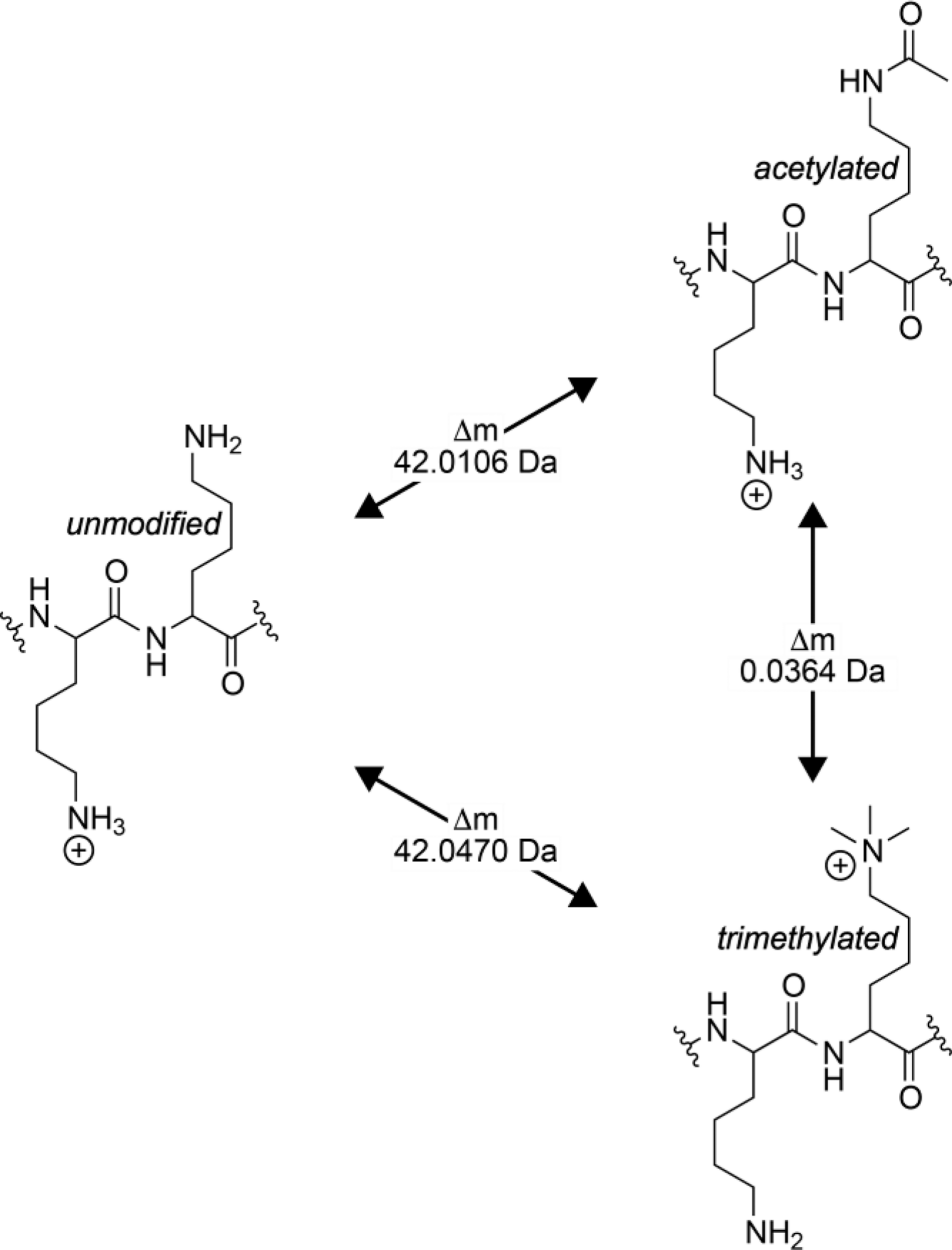
Mass Differences between Unmodified, Acetylated, and Trimethylated Fragments

Scheme 2 illustrates mass differences (Δ*m*) between unmodified, acetylated, and trimethylated fragments, according to which fragments can be separated into three different categories: 1) fragments without any modified residues, 2) fragments with mass values which are ∼42 Da higher than for a peptide without modification (i.e., 42.0106 Da for acetylation and 42.047 Da for trimethylation), and 3) fragments whose mass values differ by 36.4 mDa (trimethylation versus acetylation). We can use these three categories to study how accurately the precursor and fragment ions can be correlated in 2D MS.

**Figure 2:**
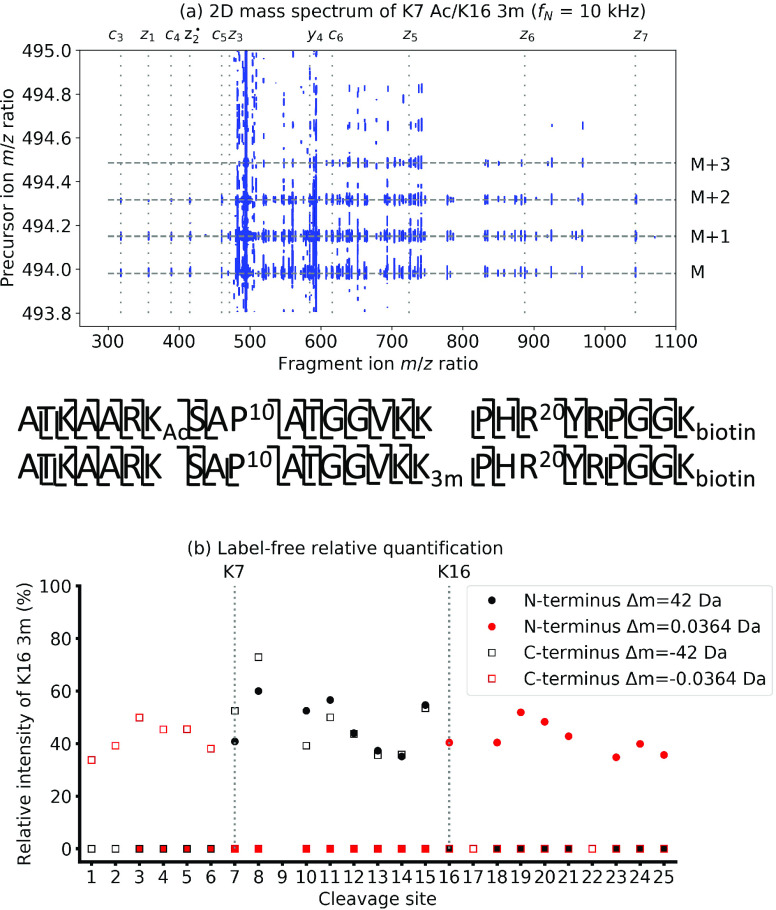
(a) Phase-corrected absorption mode 2D mass spectrum of the K7 Ac/K16 3m histone peptide mixture and sequence coverage of the two peptides. (b) Label-free relative quantification of the two histone peptides using the relative abundances of their fragment ion peaks.

Figure 2a shows the 2D mass spectrum of the [M+6H]^6+^ ions of the equimolar mixture of K7 Ac and K16 3m with a frequency range of 10 kHz in phase-corrected absorption mode. A previous study has shown that the isotopic pattern of [M+6H]^6+^ ions of methylated histone peptides is baseline-resolved in the vertical precursor ion dimension.^34^ The frequency difference between two adjacent data points (or resolution) in the vertical dimension is 4.9 Hz. Moreover, the maximum value of *t*_1_ in this experiment is 51.2 ms, which means that the minimum frequency resolution of each peak is 19.5 Hz. Therefore, the signals from the two histone peptide ions, which have a modulation frequency difference of 2.7 Hz, cannot be resolved.

The fragment ion scans for the M, M+1, M+2, and M+3 isotopes were extracted from the 2D mass spectrum and added up to reconstitute an “added-up spectrum” that is equivalent to a standard tandem mass spectrum with quadrupole isolation of the entire isotopic distribution of the precursor (see Figure S2 in the Supporting Information). Because in narrowband mode 2D MS the fragment ion scans of the isotopes are not adjacent to each other (unlike the fragment ion scans of the different isotopes in a broadband 2D mass spectrum), the noise signals are not correlated between them. Adding up the fragment ion scans therefore increased the signal-to-noise ratio in the added-up spectrum.

We peak-picked the added-up spectrum and assigned the fragment ion peaks, which lead to a 96% sequence coverage for each histone peptide, as shown in Figure 2 (the full list of assigned fragment peaks is shown in Table S1 of the Supporting Information).

Figure 2b shows the label-free relative quantification of the two histone peptides using the relative intensities in the added-up spectrum. In a previous study, 2D MS was used for label-free relative quantification of histone peptides with 0–3 methylations on the K7 residue.^35^ Their *m*/*z* ratios were clearly separated in the vertical precursor ion dimension, which made information on the difference in *m*/*z* ratios between fragment ion peaks redundant. Here we cannot resolve the two histone peptides in the vertical precursor ion dimension, so the information provided by fragment ion *m*/*z* ratios is necessary. Therefore, we calculated and plotted the results of the label-free relative quantification in the same way as in standard tandem mass spectrometry, by using the mass differences between the fragments ions of the two histone peptides (horizontal fragment ion dimension).^43^ As illustrated in Figure 2b, the information that one histone peptide is acetylated at K7 and the other trimethylated at K16 was recovered.

To determine parameters for accurate correlation between fragments of K7 Ac and K16 3m and their precursors, we acquired 2D mass spectra with frequency ranges varying between 1 and 10 kHz, with all other parameters remaining identical. As mentioned before, the spectrum resolution (or Hz per point) in the vertical precursor ion dimension is 4.9 Hz for a 10 kHz frequency range. The spectrum resolution is 2.0 Hz for a 4 kHz frequency range, 1.0 Hz for a 2 kHz frequency range, and 0.5 Hz for a 1 kHz frequency range. Furthermore, the maximum value of *t*_1_ is 128 ms for a 4 kHz frequency range, leading to a minimum peak resolution of 7.8 Hz. For a 2 kHz frequency range, the minimum peak resolution is 3.9 Hz, and 2 Hz for a 1 kHz frequency range. Since the difference in modulation frequency between the two precursor ions is 2.7 Hz, we expect the correlation between precursor and fragment ion peaks to improve with narrowing frequency ranges. Table 2 illustrates how the performance of the analysis evolved with the narrowing frequency range.

**Table 2:** Comparison in Performance between 2D Mass Spectra with Narrowing Frequency Ranges

Frequency range (kHz)	Experiment duration (min)	max. *t*_1_ (ms)	Vertical resolving power (*z*_5_ fragment)	Signal-to-noise ratio (*z*_5_ fragment)	Vertical resolving power (*z*(K7Ac)_13_^3+^ fragment)	Signal-to-noise ratio (*z*(K7Ac)_13_^3+^ fragment)	Number of assigned fragments
10	23.63	51.2	13,700	94	13,700	60	132
4	24.28	128	22,500	60	22,500	66	121
2	25.35	256	27,400	34	33,000	35	83
1	27.56	512	27,400	11	N/A	N/A	84

Table 2 shows that the total duration of the experiment increased by almost 4 min by reducing the frequency range from 10 kHz to 1 kHz. The duration *T* of the acquisition of a 2D MS data set can be expressed as
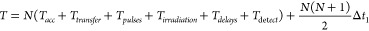
1in which *T*_*acc*_ is the total duration of the accumulation of the ions in the mass spectrometer, *T*_*transfer*_ the duration of ion transfer through the ion optics, *T*_*irradiation*_ the duration of fragmentation, *T*_*pulses*_ the duration of all the pulses applied on the excitation plates, *T*_*delays*_ the total duration of instrument delays, *T*_*detect*_ the length of the transient, and *N* is the number of increments of the encoding delay Δ*t*_1_ (see Scheme 1). The sum of these durations is on the order of 1 s. The increment Δ*t*_1_ is almost on the order of 1 ms and increases with *N*^2^ (or in 0(*N*^2^)) and should be considered for experiment design and sample consumption.

**Figure 3:**
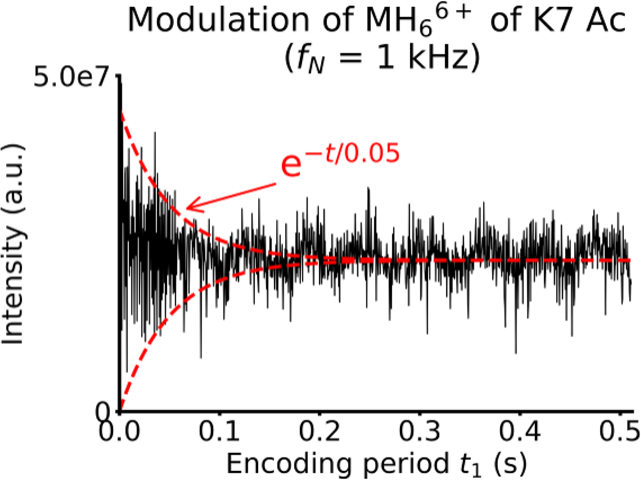
Evolution of the intensity of the [M+6H]^6+^ precursor ion of the K7 Ac histone peptide with the encoding period *t*_1_ after data processing in the horizontal dimension, extracted from the 2D mass spectrum with a 1 kHz frequency range (black). Theoretical exponential decay functions with a decay rate of 20 s^–1^ (red).

Figure 3 shows the evolution of the intensity of the precursor ion peak of the K7 Ac histone peptide (monoisotopic peak at *m*/*z* 493.9466) with the encoding delay *t*_1_ extracted from the 2D mass spectrum with a 1 kHz frequency range. The modulation of the precursor ion radius is observed through the variation of the intensity of the peak, which decreases rapidly when *t*_1_ increases. In red dashed lines, we superimposed an exponential decay with a half-life of 50 ms, which corresponds to the envelope of the intensity of the precursor. Therefore, we can estimate that the decay rate of the ion packet has a characteristic time of approximately 50 ms.

Theoretical studies and particle-in-cell simulations have shown that various factors (e.g., Coulombic repulsion or electric fields from the ICR cell electrodes) cause loss of coherent ion motion.^44−46^ In the 2D MS experiments presented here, narrowing the frequency range in the vertical dimension results in longer encoding delays after the first encoding pulse (see Scheme 1).

Loss of coherent ion motion is likely to happen when *t*_1_ is increased. Precursor ion radius modulation is heavily dependent on the coherence of the ion packet at the end of the encoding delay, and therefore decreases in amplitude when *t*_1_ increases too much.^40^ This phenomenon explains why the signal-to-noise ratio decreases with narrower frequency ranges and why the vertical resolving power fails to increase as expected. An increase in pressure during the experiment would also lead to loss of coherence for ion packets, but this would be dependent on experiment duration and not on *t*_1_, and would be evident from peak broadening in the horizontal fragment ion dimension.

**Figure 4:**
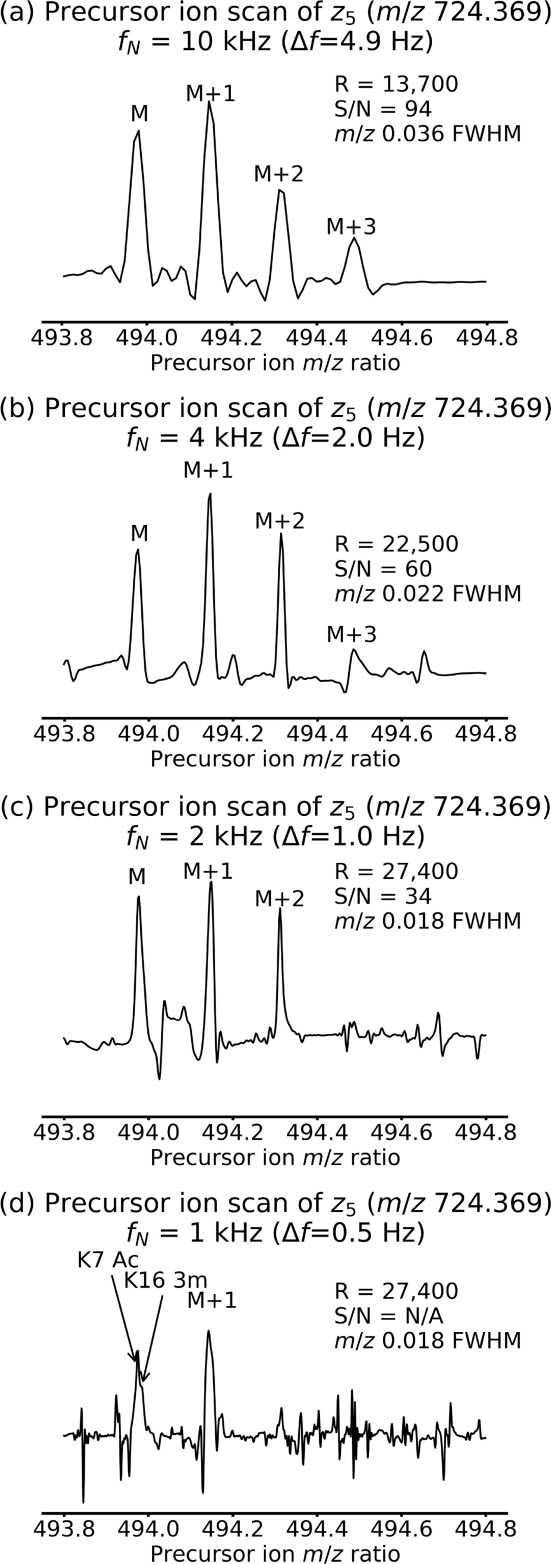
Precursor ion scans for fragment *z*_5_ of both K7 Ac and K16 3m histone peptide extracted from 2D mass spectra with *f*_N_ = (a) 10 kHz frequency range, (b) 4 kHz frequency range, (c) 2 kHz frequency range, and (d) 1 kHz frequency range. R: resolving power, S/N: signal-to-noise ratio, FWHM: full width at half-maximum.

Both K7 Ac and K16 3m have the same *z*_5_ fragment (see Figure 1 and Scheme 2). Figure 4 shows the precursor ion scans for *z*_5_ for all four 2D mass spectra. The resolution of the peaks goes from *m*/*z* 0.036 mDa at 10 kHz (Figure 4a) to *m*/*z* 0.022 at 4 kHz (Figure 4b) and *m*/*z* 0.018 at 2 kHz (Figure 4c).^42^ In the 2D mass spectrum with a 1 kHz frequency range (Figure 4d), the peaks for the K7 Ac and the K16 3m precursors start to become resolved. For the 1 kHz frequency range, the spectrum resolution in the frequency domain is 0.5 Hz, while the difference in frequency modulation between the two precursors is 2.7 Hz. Therefore, in Figure 4d the peaks are sufficiently well-defined to allow them to be both detected. However, Figure 4 also illustrates that the signal-to-noise decreases with the frequency range, as has been discussed previously: in Figure 4a and Figure 4b, four isotopic peaks of the precursor ions can be detected, but in Figure 4c only three are detected and in Figure 4d only two are detected. In Figure 4d, the noise structure is characteristic of residual noise after SANE denoising.^24^

**Figure 5:**
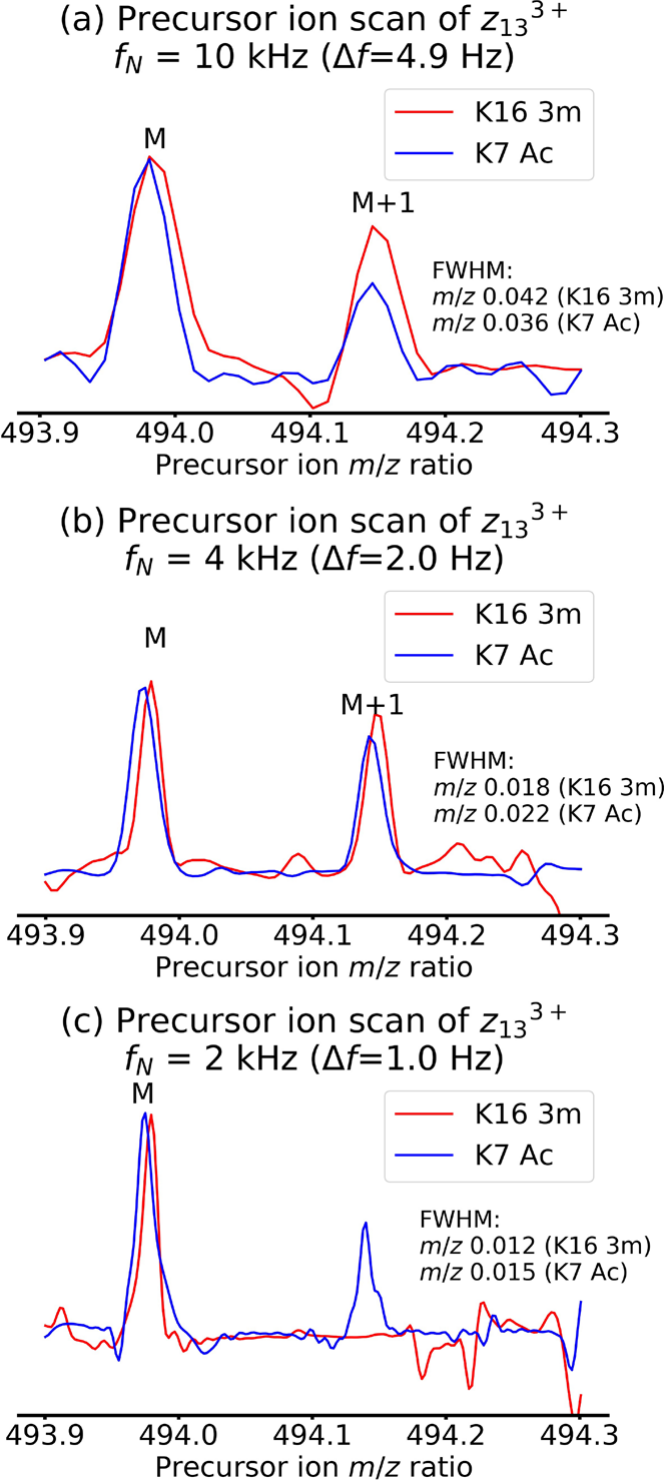
Normalized precursor ion scans for fragment *z*_13_^3+^ of both K7 Ac (*m*/*z* 563.978366) and K16 3m (*m*/*z* 577.996078) histone peptide extracted from 2D mass spectra with (a) 10 kHz frequency range, (b) 4 kHz frequency range, and (c) 2 kHz frequency range.

Fragment *z*_13_^3+^ is modified for the K16 3m peptide, but not for K7 Ac. In each 2D mass spectrum, the two vertical precursor ion scans are separated by *m*/*z* 14.0157, and they can therefore be used to examine the separation between the signals from the two histone peptides (see Figure 1 and Scheme 2). Figure 5 shows the normalized precursor ion scans of the *z*_13_^3+^ fragment of both histone peptides extracted from the 2D mass spectra with 10 kHz (Figure 5a), 4 kHz (Figure 5b), and 2 kHz (Figure 5c) precursor ion frequency range. This fragment ion was not detected at a sufficient signal-to-noise ratio for either histone peptide in the 2D mass spectrum with a precursor ion frequency range of 1 kHz.

When comparing Figure 5a–c, we see that the vertical FWHM of the peaks decreases from approximately *m*/*z* 0.039 for a frequency range of 10 kHz to *m*/*z* 0.014 for 2 kHz. This decrease is consistent with the decrease in frequency range. However, the vertical FWHM decrease by a factor of 2.6 instead of 5 as expected from the decrease in frequency ranges. We also notice a reduction in the signal-to-noise ratio from Figure 5a–c: In Figures 5a and 4b, the M+1 isotopic peak is detected, but not in Figure 5c (see also Table 2). These observations are observed in the vertical precursor ion dimension, which reflects behavior as a function of *t*_1_, and are consistent with loss of coherence in ion motion during the encoding delay as discussed above.

Although the peaks are too broad to be properly resolved, we notice that the peak for K16 3m is consistently shifted to higher *m*/*z* ratios compared to K7 Ac. There is more overlap between the two peaks in Figure 5a than in Figure 5b,c. In Figure 5b,c, this result is consistent with the difference in modulation frequency between the two histone peptides, which is 2.7 Hz.

The M+1 isotopic peak is detected for both histone peptides in Figure 5a,b, and the shift in *m*/*z* ratio between the two precursor ion scans is also evident. In the M+1 isotopic peak, the contribution of ^2^H, ^15^N, ^17^O, and ^33^S (the sulfur is in the biotinylation) is about 13% of the total abundance of the M+1 peak and corresponds to a mass difference of over 9 mDa (see Tables S5 and S6 in the Supporting Information). Therefore, the M+1 isotopic peak corresponds to multiple isotopologues (^13^C, ^2^H, ^15^N, ^17^O, ^33^S) with mass differences that are of the same order of magnitude as the mass difference between K7 Ac and K16 3m, instead of one isotopologue (^12^C, ^1^H, ^14^N, ^16^O, ^32^S), which is a cause of peak shift. Therefore, using the M+1 isotopic peak to detect the peak shift between K7 Ac and K16 3m is not as accurate as using the monoisotopic peak.

**Figure 6:**
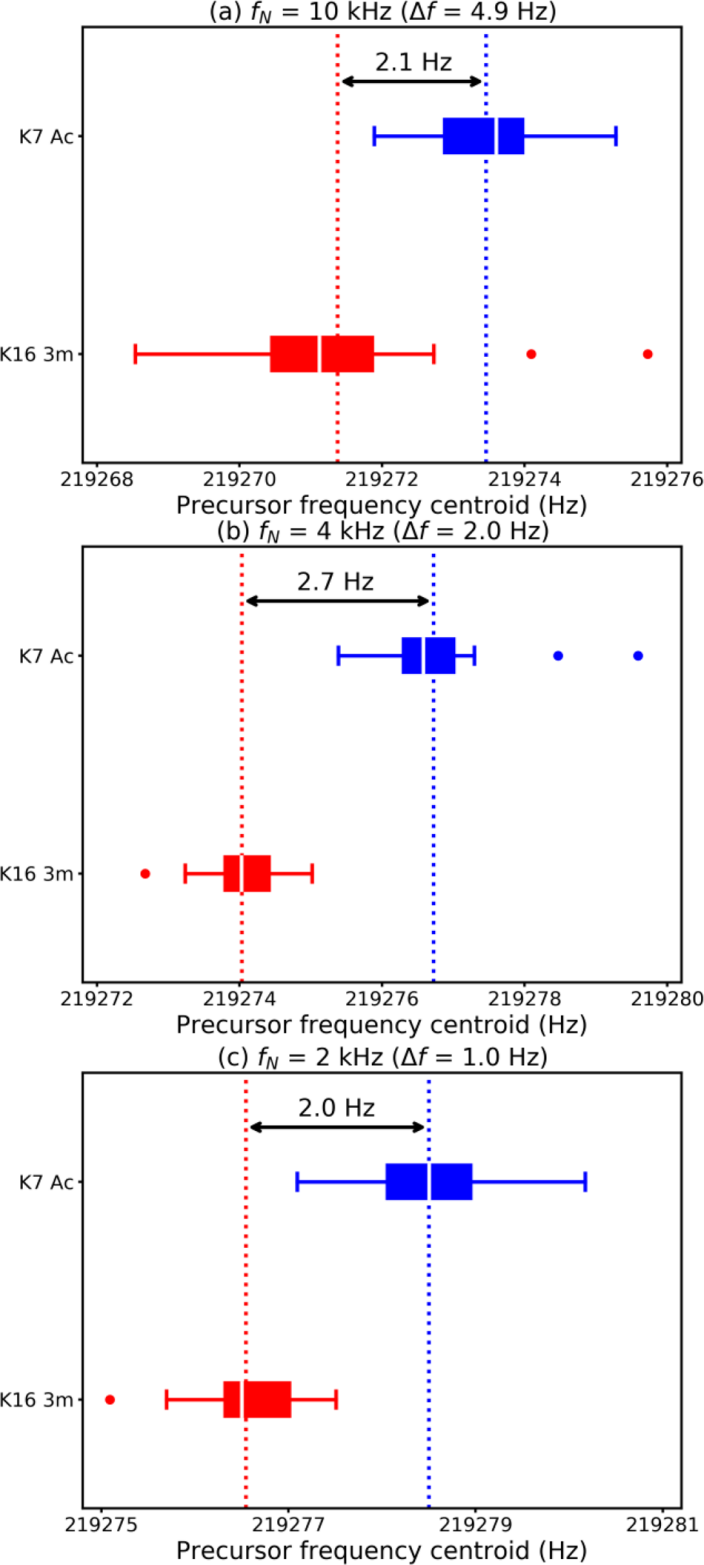
Distribution of frequencies of fragment ion peaks (vertical precursor ion dimension) after Lorentzian fit for fragment ions *c*_7–15_ and *y/z*_11–18_ of the K16 3m (red) and the K7 Ac (blue) histone peptides (M isotopic peaks only). The vertical dotted lines indicate the average frequency (arithmetic mean) measured for the fragments of K16 3m (red) and K7 Ac (blue). The medians are indicated in white in the boxplots. These measurements were performed on the 2D mass spectra acquired with (a) 10 kHz frequency range, (b) 4 kHz frequency range, and (c) 2 kHz frequency range.

Figure 6 shows box-plot representations of the positions of the fragment ion peaks in the vertical precursor ion scans (in the frequency domain) after a Lorentzian linefit for the *c*_7–15_ and *y*/*z*_11–18_ fragments, for both histone peptides, extracted from the 2D mass spectrum with a 10 kHz (Figure 6a), 4 kHz (Figure 6b), and 2 kHz (Figure 6c) frequency range.

Between the plots, the frequency range of the peaks shifts by approximately 6 Hz (219,268–219,276 Hz in Figure 6a and 219,275–219,281 Hz in Figure 6c). This variation is due to the fact that the lowest frequency in the excitation pulses was adjusted for each 2D MS experiment (see Table 1), and there is a slight imprecision in the Bruker metadata.^35^ We also notice that the number of data points decreases when the frequency range becomes narrower, which is consistent with the decrease in signal-to-noise with narrowing frequency range shown in Table 2.

The vertical frequencies measured for the fragments of K16 3m and K7 Ac form two distinct populations centered around average frequencies that differ by 2.1 Hz for a 10 kHz frequency range, 2.7 Hz for a 4 kHz frequency range, and 2.0 Hz for a 2 kHz frequency range, which is consistent with the theoretical difference in cyclotron frequency between the [M+6H]^6+^ ions of the two histone peptides (see Figure 1). For the 2D mass spectra with a 10 kHz and a 2 kHz frequency range, there is a partial overlap between the fragments of K16 3m and the fragments of K7 Ac, but not for the 2D mass spectrum with a 4 kHz frequency range. This result is likely due to two competing factors. On the one hand, narrowing the frequency range improves the accuracy of the frequency measurement because the data points are closer to each other. On the other hand, the reduction in signal-to-noise ratio, which is caused by longer values of the encoding delay *t*_1_ (see Scheme 1), induces errors in the frequency measurement.

The same analysis was performed for the *c*_16–25_ and *y*/*z*_19–25_ fragments of the M isotopes of the two histone peptides (see Figure S6 in the Supporting Information), which are only separated by 0.0364 Da horizontally (see Figure 1), and for the *c*_7–15_ and *y*/*z*_11–18_ fragments of the M+1 isotopes of the two histone peptides (see Figure S7 in the Supporting Information), in which multiple isotopologues overlap. In both cases, the two ion populations could not be distinguished properly, which shows that peaks overlapping causes errors in the measurement.

## Conclusion

In this study, we performed phase-corrected narrowband 2D MS on the mixture of an acetylated histone peptide and a trimethylated histone peptide. With a 10 kHz frequency range in the vertical precursor ion dimension, the distance between data points in the vertical dimension of the final 2D mass spectrum was nearly twice the difference in cyclotron frequency between the two precursor ions. Nevertheless, we not only achieved nearly complete sequence coverage of both histone peptides and relative quantification of the modifications but also managed nearly complete correlation of fragments with their precursor ions. Narrowing the frequency range in the vertical dimension, with all other parameters remaining equal, made the correlation more accurate, but only to a certain point. Narrowing the frequency range lead to longer encoding delays in the pulse sequence to the point where ion packets lost coherence. As a result, the precursor ion radius modulation was decreased, and therefore the signal-to-noise ratio of the fragment ion peaks in the 2D mass spectrum decreased.

Until now, the vertical resolving power has been used as a measure of the accuracy of the precursor-fragment correlation in 2D MS. This study shows that the vertical resolving power underestimates the precursor-fragment correlation. Our new results show that a more accurate measurement of the precursor-fragment correlation is less than the difference between two data points in the vertical dimension.

To increase the precursor-fragment correlation even further, ICR cells that have been designed to keep ion packets coherent for longer time periods, such as the electrically compensated or the dynamically harmonized cell, can be used, as has already successfully been done in 1D FT-ICR MS.^47−50^ We can also increase precursor-fragment correlation by decreasing the minimum distance between data points nonuniform sampling.^24^ Finally, we can move beyond the Fourier transform in the vertical dimension for data processing methods that yield more accurate frequency measurements, such as the filter diagonalization method.^51−53^ This study shows that 2D MS is a useful analytical tool for tandem mass spectrometry studies of isobaric species with the same charge state. Beyond modified peptides and proteins, 2D MS is shown here to be a viable alternative for the structural analysis of environmental samples, for which the isolation of a single ion species can be extremely challenging.^5^ Recording 2D mass spectra for different segments of the precursor ion *m*/*z* range can lead to accurate structural information of every analyte in a sample with a high degree of confidence, even if the precursor ions are not resolved in the vertical dimension.

## Data Availability

All raw data files available at: https://zenodo.org/record/7741445#.ZBNIAHbMLGh.

## Supporting Information Available

Detailed explanation of 2D MS; data processing for all 2D MS spectra; baseline correction of fragment ion scans; addition of fragment ion scans and peak-picking; baseline correction of precursor ion scans; peak-picking; and line-fitting of peaks; mass spectrum of the equimolar mixture of the acetylated histone peptide (K7) and the trimethylated histone peptide (K16) in phase-corrected absorption mode; illustration of the process of adding-up horizontal fragment ions scans; peak assignment of all 2D mass spectra theoretical mass and relative abundance of the M and M+1 isotopes of the K7 Ac and K16 3m histone peptides; distribution of frequencies of fragment ion peaks (vertical precursor ion dimension) after Lorentzian fit for fragment ions *c*_16–25_ and *y/z*_19–25_ of the K16 3m (red) and the K7 Ac (blue) histone peptides (M isotopic peaks only); distribution of frequencies of fragment ion peaks (vertical precursor ion dimension) after Lorentzian fit for fragment ions *c*_7–15_ and *y/z*_11–18_ of the K16 3m (red) and the K7 Ac (blue) histone peptides (M+1 isotopic peaks only) (PDF)
