# Supplementary material for: Ultra-Accurate Correlation between Precursor and Fragment Ions in Two-Dimensional Mass Spectrometry: Acetylated vs Trimethylated Histone Peptides
Source: J Am Soc Mass Spectrom. 2023 Mar 17;34(4):608–16. doi: 10.1021/jasms.2c00319 (PMC10080674; doi:10.1021/jasms.2c00319)
Supplement: Supplementary file 1 — js2c00319_si_001.pdf [file js2c00319_si_001.pdf]

Ultra-Accurate Correlation Between Precursor and Fragment Ions in Two-dimensional Mass Spectrometry: Acetylated vs. Trimethylated Histone Peptides Supporting Information

Michael Palasser[1], Sarah V. Heel[1], Marc-André Delsuc[2,3], Kathrin Breuker[1], Maria A. van Agthoven[1*]

[1] Institute for Organic Chemistry. University of Innsbruck. 80/82 Innrain. 6020 Innsbruck. Austria

[2] Institut de Génétique et de Biologie Moléculaire et Cellulaire. INSERM U596. UMR 7104. Université de Strasbourg. 1 rue Laurent Fries. 67404 Illkirch-Graffenstaden. France

[3] CASC4DE. Pôle API. 300 Bd. Sébastien Grant. 67400 Illkirch-Graffenstaden. France

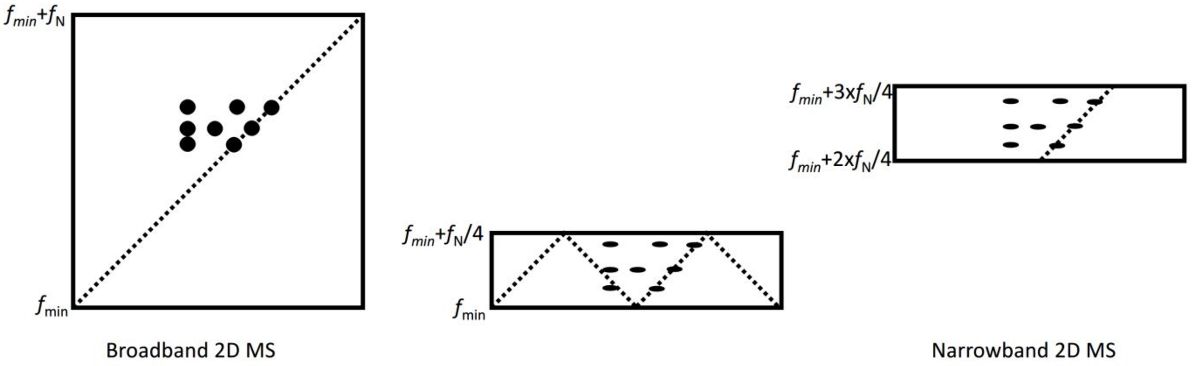

Scheme S1. Principle of narrowband 2D MS.

On Bruker FT-ICR mass spectrometers, all excitation pulses have the same frequency range. Typically, the *m/z* range of fragment ions is wider than the *m/z* range of the precursors. In broadband 2D MS, the user can control the increment of the delay t_1 (Δt_1), which determines the Nyquist frequency (f_N) of the precursor ion radius modulation, i.e. the lowest precursor ion *m/z* ratio:

$$f_N = \frac{1}{2\Delta t_1}$$

The highest precursor ion m/z ratio is set by the lowest frequency of the excitation pulse (f_{min}), which must be higher than the cyclotron frequency of the fragment with the highest *m/z* ratio.

Any precursor ion (and its fragments) whose radius modulation frequency falls outside of the frequency range defined by fmin and f_N has its signal aliased in the 2D spectrum. Scheme S1 shows what happens to the autocorrelation line when the Nyquist frequency is devided by 4: it gets folded over twice at the boundaries of the 2D spectrum (i.e. there are two foldovers). The frequency can be corrected by a simple shift in order to recover the analytical information of that *m/z* range. The resulting 2D spectrum is in narrowband. In the case shown in Scheme S1, if the number of datapoints is constant between the broadband mode 2D spectrum and the narrowband mode 2D spectrum, the resolving power has been multiplied by 4. This strategy enables the reduction of the *m/z* range of the precursor ion dimension which is useful e.g. for the top-down analysis of a single charge state of a biomolecule proteoform.

Data processing for 2D MS spectrum with Nyquist frequency 10000 Hz:

```
from spike.File import Apex
#import raw dataset:
d =
Apex.Import_2D("/Users/maartje/Desktop/donnees/21_06_15/histon
epeptide_2D_000003.d")
#set Nyquist frequency:
d.axis1.specwidth = 10000.0
#apodisation. zero-filling and Fourier transform in the horizontal fragment ion dimension:
d.apod_sin(axis=2, maxi=0.15).chsize(d.size1,
4*d.size2).rfft(axis=2)
#Digital demodulation (vertical precursor ion dimension:
d.flipphase(0.0, 180*d.axis1.htoi(74659.79), axis = 1)
#Horizontal quadratic phase-correction:
d.phaseMS(30.3, 365.1, 2041.8, 0.0, axis = 2)
#Absorption mode in the horizontal dimension:
d.real(axis=2)
#denoising. apodisation. zero-filling and Fourier transform in the vertical precursor ion
dimension:
d.sane(rank=30, axis=1).apod_sin(axis=1,
maxi=0.15).chsize(4*d.size1, d.size2).rfft(axis=1)
#Vertical linear phase-correction:
d.phaseMS(-134.5, 4.6, 0.0, 0.00, axis=1)
#Absorption mode in the vertical dimension:
d.real(axis=1)
#Frequency offset:
offset = float(d.params['FR_low'])
d.axis1.offsetfreq = 14*d.axis1.specwidth + offset
d.axis1.leftpoint = 0
d
```

Data processing for 2D MS spectrum with Nyquist frequency 4000 Hz:

```
from spike.File import Apex
d =
Apex.Import_2D("/Users/maartje/Desktop/donnees/21_06_15/histon
epeptide_2D_000004.d")
d.axis1.specwidth = 4000.0
d.apod_sin(axis=2, maxi=0.15).chsize(d.size1,
4*d.size2).rfft(axis=2)
d.flipphase(0.0, 180*d.axis1.htoi(72171.4), axis = 1)
d.phaseMS(145.6, 394.4, 2041.3, 0.0, axis = 2)
d.real(axis=2)
d. sane(rank=30, axis=1).apod_sin(axis=1,
maxi=0.15).chsize(4*d.size1, d.size2).rfft(axis=1)
d.phaseMS(21.8, 1.8, 0.0, 0.00, axis=1)
d.real(axis=1)
offset = float(d.params['FR_low'])
d.axis1.offsetfreq = 36*d.axis1.specwidth + offset
```

```
d.axis1.leftpoint = 0
d
```

Data processing for 2D MS spectrum with Nyquist frequency 2000 Hz:

```
from spike.File import Apex
d =
Apex.Import_2D("/Users/maartje/Desktop/donnees/21_06_15/histon
epeptide_2D_000005.d")
d.axis1.specwidth = 2000.0
d.apod_sin(axis=2, maxi=0.15).chsize(d.size1,
4*d.size2).rfft(axis=2)
d.flipphase(0.0, 180*d.axis1.htoi(73895.34), axis = 1)
d.phaseMS(-18.6, 374.7, 2040.5, 0.0, axis = 2)
d.real(axis=2)
d.sane(rank = 30, axis=1).apod_sin(axis=1,
maxi=0.15).chsize(4*d.size1, d.size2).rfft(axis=1)
d.phase(84.7, 0.9, 0.0, 0.00, axis=1)
d.real(axis=1)
offset = float(d.params['FR_low'])
d.axis1.offsetfreq = 72*d.axis1.specwidth + offset
d.axis1.leftpoint = 0
d
```

Data processing for 2D MS spectrum with Nyquist frequency 1000 Hz:

```
from spike.File import Apex
d =
Apex.Import_2D("/Users/maartje/Desktop/donnees/21_06_15/histon
epeptide_2D_000006.d")
d.axis1.specwidth = 1000.0
d.apod_sin(axis=2, maxi=0.15).chsize(d.size1,
4*d.size2).rfft(axis=2)
d.flipphase(0.0, 180*d.axis1.htoi(74402.99923849203), axis =
1)
d.phaseMS(-168.7, 369.3, 2039.6, 0.0, axis = 2)
d.real(axis=2)
d.sane(rank=30, axis=1).apod_sin(axis=1,
maxi=0.15).chsize(4*d.size1, d.size2).rfft(axis=1)
d.phaseMS(5.9, 0.5, 0.0, 0.00, axis=1)
d.real(axis=1)
#d.modulus()
offset = float(d.params['FR_low'])
d.axis1.offsetfreq = 144*d.axis1.specwidth + offset
d.axis1.leftpoint = 0
d
```

Baseline correction of fragment ion scans. addition of fragment ion scans. and peak-picking:

```
from spike.read_msh5 import read_msh5 as RM
from spike.plugins.bcorr import autopoints
```

```
from spike.FTICR import FTICRData
```

#load data set:

```
data =
RM('/Users/maartje/Desktop/donnees/21_06_15/histonepeptide_2D_
000004_phased_sane30.msh5')
d = data.resi(1)
```

#extract fragment ion scans:

```
A = [1556, 1517, 1479, 1441]
g = FTICRData(dim = 1, shape = d.size2, buffer = [0 for i in
range(d.size2)], name = None)
```

#baseline-correct fragment ion scans and add them up:

```
for i in A:
     q = d.row(a)
     r = q.copy()
     xp = autopoints(q, int(30000))
     r.bcorr(method='spline', xpoints=xp, nsmooth=2)
     r.set_unit('m/z').display(new_fig = False)
     g.add(r)
g.unit = "m/z"
```

#peak-picking:

```
g.pp(threshold=3.0e6)
```

#centroiding:

```
g.centroid()
g.unit = "Hz"
df = g.pk2pandas()
df.head()
```

#save peaklist for manipulation in Excel:

```
df.to_excel("/Users/maartje/Desktop/donnees/21_06_15/data
analysis/f4000/peak_list_2D_allisotopes.xlsx")
g.save_msh5("/Users/maartje/Desktop/donnees/21_06_15/data
analysis/f4000/allisotopes_spectrum_baseline_corrected.msh5")
```

Baseline correction of precursor ion scans. peak-picking. and line-fitting of peaks:

```
from spike.read_msh5 import read_msh5 as RM
from spike.plugins.bcorr import autopoints
from spike.FTICR import FTICRData
```

#load data set:

```
data =
RM('/Users/maartje/Desktop/donnees/21_06_15/histonepeptide_2D_
000004_phased_sane30.msh5')
d = data.resi(1)
```

#extract horizontal fragment ion scan:

```
f = d.row(1556)
f.unit = "points"
```

#horizontal peak-picking:

```
f.pp(threshold=3.0e6)
f.display()
f.display_peaks(peak_label=True)
```

```
df = f.pk2pandas()
#Peak-picking of vertical precursor ion scans:
for i in df['m/z']:
    if 301 < d.axis2.itomz(i) < 1500:
        b = d.col(i)       #extract vertical precursor ion scans
        xp = autopoints(b, 50)
        b.bcorr(method='spline', xpoints=xp, nsmooth=2)
     #baseline correction
        b.unit = "Hz"
        b.pp(threshold=8e5)          #peak-picking
        b.centroid()            #centroiding
        b.fit(zoom = [219000, 219300])        #Lorentzian fit
        dg = b.pk2pandas()

# save peaklist for manipulation in Excel:
dg.to_excel("/Users/maartje/Desktop/donnees/21_06_15/data
analysis/f10000/fit
13C/peak_list_2D_{}.xlsx".format(d.axis2.itomz(i)))
```

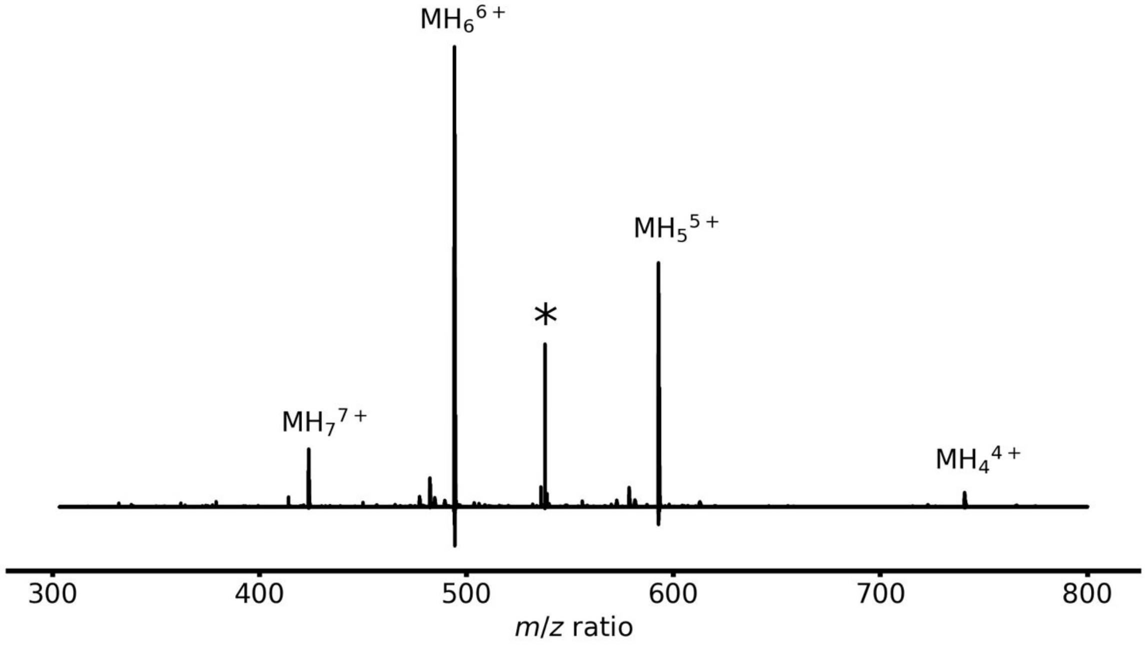

Figure S1. Mass spectrum of the equimolar mixture of the acetylated histone peptide (K7) and the trimethylated histone peptide (K16) in phase-corrected absorption mode.
* Chemical contaminant

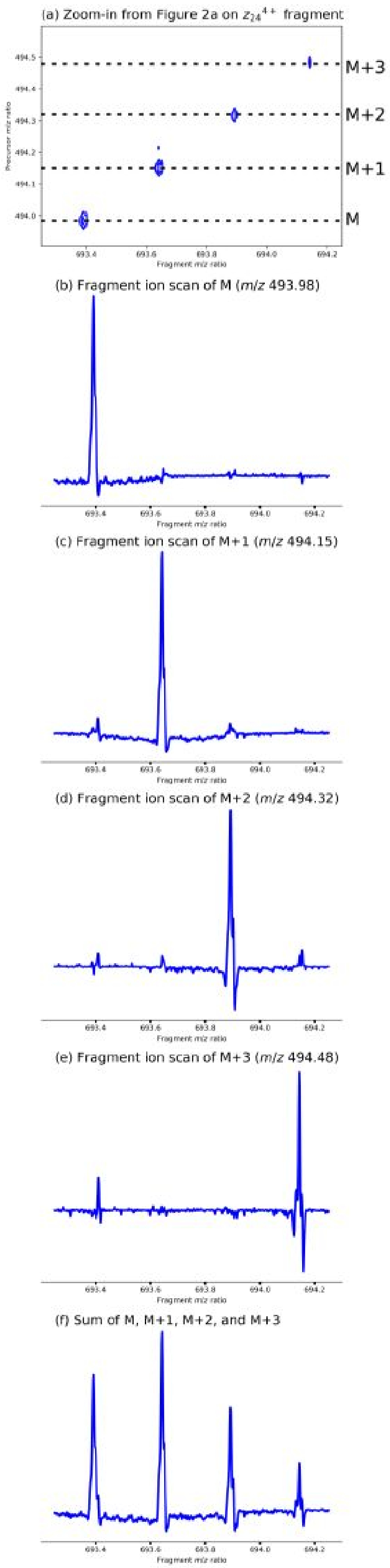
(a) Zoom-in from Figure 2a on z_{24}^{4+} fragment
M+3
M+2
M+1
M
Precursor m/z ratio
Fragment m/z ratio
(b) Fragment ion scan of M (*m/z* 493.98)
(c) Fragment ion scan of M+1 (*m/z* 494.15)
(d) Fragment ion scan of M+2 (*m/z* 494.32)
(e) Fragment ion scan of M+3 (*m/z* 494.48)
(f) Sum of M, M+1, M+2, and M+3

Figure S2. (a) Zoom-in on the 2D mass spectrum from Fig. 2a (f_N = 10kHz) on the z_{24}^{4+} fragment. (b) Fragment ion scan of the M isotope. (c) Fragment ion scan of the M+1 isotope. (d) Fragment ion scan of the M+2 isotope. (e) Fragment ion scan of the M+3 isotope. (f) Sum of the fragment ion scans of all four isotopes.

Table S1. Peak assignment of the 2D mass spectrum with f_N = 10 kHz.

***m/z* ratio**	**Assignment**	**Mass accuracy (ppm)**	**Precursor**
318.213495*	c_3	-0.27	K16 3m/K7 Ac
389.250356*	c_4	-0.87	K16 3m/K7 Ac
460.288046*	c_5	0.52	K16 3m/K7 Ac
616.389452*	c_6	0.86	K16 3m/K7 Ac
744.484272	c_7	0.52	K16 3m
372.745821	c_7^{2+}	0.65	K16 3m
831.516361	c_8	0.54	K16 3m
416.262156	c_8^{2+}	1.35	K16 3m
999.606527	c_{10}	0.74	K16 3m
500.306751	c_{10}^{2+}	0.44	K16 3m
1070.64869	c_{11}	5.41	K16 3m
535.825689	c_{11}^{2+}	1.12	K16 3m
1171.69092	c_{12}	0.29	K16 3m
586.349344	c_{12}^{2+}	0.71	K16 3m
614.860319	c_{13}^{2+}	1.07	K16 3m
643.370765	c_{14}^{2+}	0.58	K16 3m
1384.80267	c_{15}	0.54	K16 3m
692.905353	c_{15}^{2+}	1.09	K16 3m
777.976527	c_{16}^{2+}	1.25	K16 3m
518.986445	c_{16}^{3+}	0.61	K16 3m
389.492783	c_{16}^{4+}	3.51	K16 3m
594.036174	c_{18}^{3+}	1.35	K16 3m
445.778077	c_{18}^{4+}	-0.61	K16 3m
959.078989	c_{19}^{2+}	0.12	K16 3m
639.722056	c_{19}^{3+}	0.59	K16 3m
480.043278	c_{19}^{4+}	0.42	K16 3m
691.754923	c_{20}^{3+}	-0.66	K16 3m
519.069028	c_{20}^{4+}	1.30	K16 3m
746.110342	c_{21}^{3+}	0.70	K16 3m
559.834489	c_{21}^{4+}	0.54	K16 3m
830.495269	c_{23}^{3+}	0.99	K16 3m
622.870894	$c_{23}^{4+\bullet}$	0.31	K16 3m
637.126182	$c_{24}^{4+\bullet}$	0.19	K16 3m
651.633844	c_{25}^{4+}	0.70	K16 3m
357.171291*	z_1	-1.09	K16 3m/K7 Ac
373.190679	y_1	0.74	K16 3m/K7 Ac
415.201316	$z_2^{\bullet}$	0.84	K16 3m/K7 Ac
471.21498*	z_3	0.79	K16 3m/K7 Ac
487.233075	y_3	-0.52	K16 3m/K7 Ac

584.285943	y_4	-0.26	K16 3m/K7 Ac
724.368814*	z_5	0.46	K16 3m/K7 Ac
740.387712	y_5	0.68	K16 3m/K7 Ac
903.451003*	y_6	0.52	K16 3m/K7 Ac
887.431837*	z_6	0.03	K16 3m/K7 Ac
1043.53129*	z_7	-1.57	K16 3m/K7 Ac
530.278407	y_7^{2+}	-1.99	K16 3m/K7 Ac
522.269938*	z_7^{2+}	-0.31	K16 3m/K7 Ac
590.799463*	z_8^{2+}	-0.16	K16 3m/K7 Ac
647.333726	y_9^{2+}	-2.43	K16 3m/K7 Ac
1405.74003*	z_{10}	0.33	K16 3m/K7 Ac
703.373677*	z_{10}^{2+}	0.37	K16 3m/K7 Ac
469.250649	z_{10}^{3+}	-1.54	K16 3m/K7 Ac
525.96575	z_{11}^{3+}	0.78	K16 3m
558.988191	z_{12}^{3+}	0.08	K16 3m
866.992512	$z_{13}^{2+\bullet}$	-0.82	K16 3m
577.995294	z_{13}^{3+}	-0.01	K16 3m
597.002205	z_{14}^{3+}	-0.42	K16 3m
895.50236	$z_{14}^{2+\bullet}$	-1.78	K16 3m
630.68431	z_{15}^{3+}	-1.12	K16 3m
654.362089	z_{16}^{3+}	-3.00	K16 3m
515.539762	$z_{17}^{4+\bullet}$	-0.47	K16 3m
1065.58898	$z_{18}^{2+\bullet}$	-2.17	K16 3m
710.394574	z_{18}^{3+}	0.79	K16 3m
533.047072	z_{18}^{4+}	-0.48	K16 3m
739.402872	z_{19}^{3+}	-2.46	K16 3m
554.805228	z_{19}^{4+}	-0.19	K16 3m
782.437266	$z_{20}^{3+\bullet}$	-2.15	K16 3m
586.829131	z_{20}^{4+}	0.09	K16 3m
834.473338	$z_{21}^{3+\bullet}$	0.82	K16 3m
625.854495	z_{21}^{4+}	0.23	K16 3m
858.151984	$z_{22}^{3+\bullet}$	0.34	K16 3m
643.613213	z_{22}^{4+}	-0.65	K16 3m
881.831732	$z_{23}^{3+\bullet}$	1.13	K16 3m
661.37222	z_{23}^{4+}	-1.04	K16 3m
665.379334	y_{23}^{4+}	2.62	K16 3m
924.525048	$z_{24}^{3+\bullet}$	-4.33	K16 3m
693.395663	z_{24}^{4+}	-1.43	K16 3m
554.919576	z_{24}^{5+}	1.44	K16 3m
482.110118	y_{25}^{6+}	-2.37	K16 3m
575.128153	z_{25}^{5+}	-0.28	K16 3m
786.4948	c_7	0.45	K7 Ac
873.526222	c_8	-0.29	K7 Ac
437.266495	c_8^{2+}	-0.87	K7 Ac
521.311782	c_{10}^{2+}	-0.06	K7 Ac
556.830567	c_{11}^{2+}	0.35	K7 Ac

1212.69219	$c_{12}^{\bullet}$	-0.93	K7 Ac
607.354455	c_{12}^{2+}	0.40	K7 Ac
635.865258	c_{13}^{2+}	0.50	K7 Ac
664.376067	c_{14}^{2+}	0.59	K7 Ac
713.910758	c_{15}^{2+}	1.23	K7 Ac
777.957919	c_{16}^{2+}	0.72	K7 Ac
518.974198	c_{16}^{3+}	0.38	K7 Ac
890.530873	c_{18}^{2+}	-0.40	K7 Ac
594.023967	c_{18}^{3+}	1.22	K7 Ac
959.062575	c_{19}^{2+}	1.97	K7 Ac
639.710464	c_{19}^{3+}	1.43	K7 Ac
480.034438	c_{19}^{4+}	0.96	K7 Ac
691.744473	c_{20}^{3+}	1.77	K7 Ac
519.059868	c_{20}^{4+}	1.18	K7 Ac
746.096024	c_{21}^{3+}	-2.24	K7 Ac
559.82586	c_{21}^{4+}	1.38	K7 Ac
830.482861	c_{23}^{3+}	0.65	K7 Ac
622.861748	$c_{23}^{4+\bullet}$	0.24	K7 Ac
637.117893	$c_{24}^{4+\bullet}$	1.45	K7 Ac
651.625101	c_{25}^{4+}	1.25	K7 Ac
767.924953	$z_{11}^{2+\bullet}$	0.18	K7 Ac
511.949752	z_{11}^{3+}	0.12	K7 Ac
544.972364	z_{12}^{3+}	-0.24	K7 Ac
817.457861	$z_{12}^{2+\bullet}$	-1.42	K7 Ac
550.313003	y_{12}^{3+}	1.69	K7 Ac
563.979475	z_{13}^{3+}	-0.31	K7 Ac
845.96886	$z_{13}^{2+\bullet}$	-1.05	K7 Ac
874.486619	$z_{14}^{2+\bullet}$	7.02	K7 Ac
582.986954	z_{14}^{3+}	0.25	K7 Ac
616.668764	z_{15}^{3+}	-0.98	K7 Ac
640.348505	z_{16}^{3+}	0.16	K7 Ac
696.376373	z_{18}^{3+}	-2.86	K7 Ac
522.535775	z_{18}^{4+}	0.35	K7 Ac
544.293759	z_{19}^{4+}	0.30	K7 Ac
782.426437	$z_{20}^{3+\bullet}$	-0.49	K7 Ac
586.820439	z_{20}^{4+}	0.78	K7 Ac
834.45899	$z_{21}^{3+\bullet}$	-1.84	K7 Ac
625.846285	z_{21}^{4+}	1.64	K7 Ac
858.138414	$z_{22}^{3+\bullet}$	-1.34	K7 Ac
643.605215	z_{22}^{4+}	1.05	K7 Ac
881.481599	z_{23}^{3+}	-1.20	K7 Ac
661.364125	z_{23}^{4+}	0.47	K7 Ac
693.387879	z_{24}^{4+}	0.47	K7 Ac
554.912305	z_{24}^{5+}	1.45	K7 Ac
718.645634	z_{25}^{4+}	-5.34	K7 Ac

575.119383	z_{25}^{5+}	-2.87	K7 Ac
482.105227	y_{25}^{6+}	0.07	K7 Ac

* Peak used for internal calibration

Average of absolute value of mass accuracy: 1 ppm

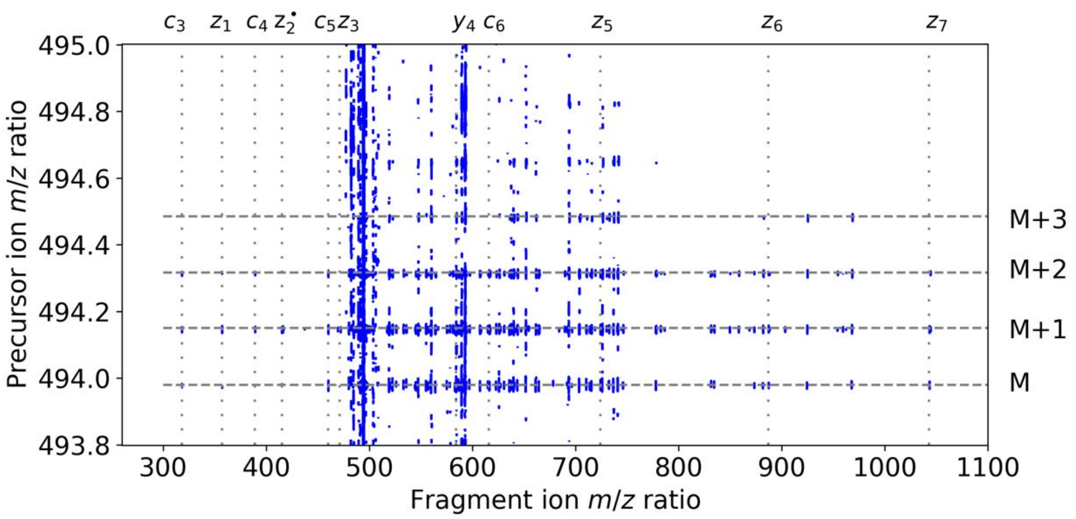

Figure S3. 2D mass spectrum of the K7 Ac/K16 3m histone peptide mixture with a frequency range of 4 kHz.

Table S2. Peak assignment of the 2D mass spectrum with f_N = 4 kHz.

***m/z* ratio**	**Assignment**	**Mass accuracy (ppm)**	**Precursor**
318.213261*	c_3	-1.01	K16 3m/K7 Ac
389.250361*	c_4	-0.86	K16 3m/K7 Ac
460.287926*	c_5	0.26	K16 3m/K7 Ac
616.389939	c_6	1.65	K16 3m/K7 Ac
744.483895*	c_7	0.02	K16 3m
831.516757*	c_8	1.02	K16 3m
416.262379	c_8^{2+}	1.89	K16 3m
500.30768	c_{10}^{2+}	2.30	K16 3m
535.825631	c_{11}^{2+}	1.01	K16 3m
1171.68885	c_{12}	-1.48	K16 3m
586.349702	c_{12}^{2+}	1.32	K16 3m
614.860268	c_{13}^{2+}	0.99	K16 3m
643.371243	c_{14}^{2+}	1.32	K16 3m
692.905747	c_{15}^{2+}	1.66	K16 3m
777.97488	c_{16}^{2+}	-0.87	K16 3m
518.986528	c_{16}^{3+}	0.77	K16 3m
389.491774	c_{16}^{4+}	0.92	K16 3m
594.035947	c_{18}^{3+}	0.97	K16 3m
445.777954	c_{18}^{4+}	-0.88	K16 3m
959.07772	c_{19}^{2+}	-1.20	K16 3m
639.722457	c_{19}^{3+}	1.22	K16 3m
480.043738	c_{19}^{4+}	1.38	K16 3m
691.756277	c_{20}^{3+}	1.30	K16 3m
519.068961	c_{20}^{4+}	1.17	K16 3m
746.11024	c_{21}^{3+}	0.56	K16 3m
559.834651	c_{21}^{4+}	0.83	K16 3m
830.494731	c_{23}^{3+}	0.34	K16 3m

622.872165	$c_{23}^{4+\bullet}$	2.36	K16 3m
849.490392	c_{24}^{3+}	-13.20	K16 3m
637.125676	$c_{24}^{4+\bullet}$	-0.61	K16 3m
651.634126	c_{25}^{4+}	1.14	K16 3m
357.17198*	z_1	0.84	K16 3m/K7 Ac
373.190527	y_1	0.33	K16 3m/K7 Ac
415.200752*	$z_2^{\bullet}$	-0.52	K16 3m/K7 Ac
472.222866*	$z_3^{\bullet}$	0.92	K16 3m/K7 Ac
740.388083	y_5	1.19	K16 3m/K7 Ac
724.368673*	z_5	0.26	K16 3m/K7 Ac
903.450611*	y_6	0.09	K16 3m/K7 Ac
887.430963*	z_6	-0.95	K16 3m/K7 Ac
1043.53017	z_7	-2.64	K16 3m/K7 Ac
522.270551*	z_7^{2+}	0.87	K16 3m/K7 Ac
590.800316	z_8^{2+}	1.29	K16 3m/K7 Ac
647.334494*	y_9^{2+}	-1.24	K16 3m/K7 Ac
1405.73964*	z_{10}	0.06	K16 3m/K7 Ac
711.383249	y_{10}^{2+}	0.66	K16 3m/K7 Ac
703.373679*	z_{10}^{2+}	0.37	K16 3m/K7 Ac
469.251501	z_{10}^{3+}	0.28	K16 3m/K7 Ac
525.96569	z_{11}^{3+}	0.66	K16 3m
838.48053	$z_{12}^{2+\bullet}$	-2.34	K16 3m
558.988574	z_{12}^{3+}	0.76	K16 3m
577.994988	z_{13}^{3+}	-0.54	K16 3m
895.503996	$z_{14}^{2+\bullet}$	0.04	K16 3m
597.002892	z_{14}^{3+}	0.73	K16 3m
630.684798	z_{15}^{3+}	-0.34	K16 3m
654.364394	z_{16}^{3+}	0.52	K16 3m
710.393827	z_{18}^{3+}	-0.26	K16 3m
533.046214	z_{18}^{4+}	-2.09	K16 3m
739.40385	z_{19}^{3+}	-1.13	K16 3m
782.438587	$z_{20}^{3+\bullet}$	-0.47	K16 3m
586.829597	z_{20}^{4+}	0.89	K16 3m
834.472746	$z_{21}^{3+\bullet}$	0.11	K16 3m
625.855107	z_{21}^{4+}	1.20	K16 3m
643.614232	z_{22}^{4+}	0.93	K16 3m
881.831324	$z_{23}^{3+\bullet}$	0.67	K16 3m
661.372904	z_{23}^{4+}	-0.01	K16 3m
665.377113	y_{23}^{4+}	-0.72	K16 3m
529.29915	z_{23}^{5+}	-1.20	K16 3m
693.39705	z_{24}^{4+}	0.57	K16 3m
554.918895	z_{24}^{5+}	0.21	K16 3m
718.657276	z_{25}^{4+}	-1.80	K16 3m
575.329986	$z_{25}^{5+\bullet}$	0.19	K16 3m
786.494522	c_7	0.10	K7 Ac

873.526144*	c_8	-0.38	K7 Ac
437.267311	c_8^{2+}	1.00	K7 Ac
521.312279	c_{10}^{2+}	0.89	K7 Ac
1112.65088	c_{11}	-2.33	K7 Ac
556.830709	c_{11}^{2+}	0.60	K7 Ac
607.354687	c_{12}^{2+}	0.78	K7 Ac
1269.7077	$c_{13}^{\bullet}$	-5.58	K7 Ac
635.865291	c_{13}^{2+}	0.55	K7 Ac
664.376413	c_{14}^{2+}	1.11	K7 Ac
713.910826	c_{15}^{2+}	1.32	K7 Ac
777.958452	c_{16}^{2+}	1.40	K7 Ac
518.974301	c_{16}^{3+}	0.58	K7 Ac
594.023769	c_{18}^{3+}	0.89	K7 Ac
959.06333	c_{19}^{2+}	2.76	K7 Ac
639.710389	c_{19}^{3+}	1.32	K7 Ac
480.040237	c_{19}^{4+}	13.04	K7 Ac
691.744576	c_{20}^{3+}	1.92	K7 Ac
519.059519	c_{20}^{4+}	0.50	K7 Ac
746.099626	c_{21}^{3+}	2.59	K7 Ac
559.825909	c_{21}^{4+}	1.47	K7 Ac
622.862944	$c_{23}^{4+\bullet}$	2.15	K7 Ac
637.118485	$c_{24}^{4+\bullet}$	2.38	K7 Ac
849.490392	c_{24}^{3+}	1.08	K7 Ac
651.626003	c_{25}^{4+}	2.63	K7 Ac
511.949819	z_{11}^{3+}	0.25	K7 Ac
517.29024	y_{11}^{3+}	1.88	K7 Ac
544.971619	z_{12}^{3+}	-1.61	K7 Ac
845.971517	$z_{13}^{2+\bullet}$	2.09	K7 Ac
563.979789	z_{13}^{3+}	0.24	K7 Ac
874.480376	$z_{14}^{2+\bullet}$	-0.12	K7 Ac
582.986783	z_{14}^{3+}	-0.04	K7 Ac
616.669766	z_{15}^{3+}	0.65	K7 Ac
640.348458	z_{16}^{3+}	0.09	K7 Ac
696.376934	z_{18}^{3+}	-2.05	K7 Ac
522.534673	z_{18}^{4+}	-1.76	K7 Ac
544.293585	z_{19}^{4+}	-0.02	K7 Ac
586.820545	z_{20}^{4+}	0.96	K7 Ac
782.425578	$z_{20}^{3+\bullet}$	-1.59	K7 Ac
834.460864	$z_{21}^{3+\bullet}$	0.40	K7 Ac
625.84625	z_{21}^{4+}	1.58	K7 Ac
858.14154	$z_{22}^{3+\bullet}$	2.30	K7 Ac
643.605599	z_{22}^{4+}	1.65	K7 Ac
881.819235	$z_{23}^{3+\bullet}$	0.72	K7 Ac
661.363721	z_{23}^{4+}	-0.14	K7 Ac
693.387822	z_{24}^{4+}	0.39	K7 Ac

554.911386	z_{24}^{5+}	-0.20	K7 Ac
718.89977	$z_{25}^{4+\bullet}$	-2.31	K7 Ac
575.119372	z_{25}^{5+}	-2.89	K7 Ac
578.323453	y_{25}^{5+}	-2.30	K7 Ac

* Peak used for internal calibration
Average of absolute value of mass accuracy: 1.3 ppm

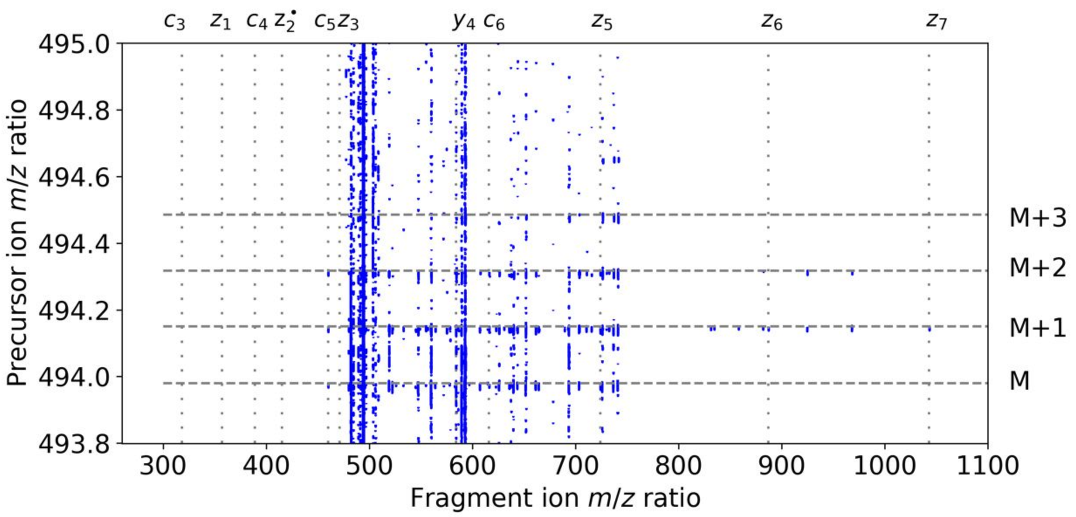

Figure S4. 2D mass spectrum of the K7 Ac/K16 3m histone peptide mixture with a frequency range of 2 kHz.

Table S3. Peak assignment of the 2D mass spectrum with f_N = 2 kHz.

***m/z* ratio**	**Assignment**	**Mass accuracy (ppm)**	**Precursor**
318.213195*	c_3	-1.21	K16 3m/K7 Ac
389.25042*	c_4	-0.71	K16 3m/K7 Ac
460.288063*	c_5	0.55	K16 3m/K7 Ac
616.389387*	c_6	0.76	K16 3m/K7 Ac
744.484525*	c_7	0.86	K16 3m
831.515355*	c_8	-0.67	K16 3m
416.262046	c_8^{2+}	1.09	K16 3m
500.307216	c_{10}^{2+}	1.37	K16 3m
535.825536	c_{11}^{2+}	0.83	K16 3m
586.349508	c_{12}^{2+}	0.99	K16 3m
614.860912	c_{13}^{2+}	2.04	K16 3m
643.370943	c_{14}^{2+}	0.86	K16 3m
692.906565	c_{15}^{2+}	2.84	K16 3m
518.986246	c_{16}^{3+}	0.22	K16 3m
389.492784	c_{16}^{4+}	3.51	K16 3m
594.036103	c_{18}^{3+}	1.23	K16 3m
639.721878	c_{19}^{3+}	0.32	K16 3m
480.043726	c_{19}^{4+}	1.35	K16 3m
519.069441	c_{20}^{4+}	2.09	K16 3m
559.834847	c_{21}^{4+}	1.18	K16 3m
622.869897	$c_{23}^{4+\bullet}$	-1.29	K16 3m
637.12539	$c_{24}^{4+\bullet}$	-1.06	K16 3m
651.633764	c_{25}^{4+}	0.58	K16 3m
357.171941*	z_1	0.73	K16 3m/K7 Ac
415.201326*	$z_2^{\bullet}$	0.86	K16 3m/K7 Ac
472.22214*	$z_3^{\bullet}$	-0.62	K16 3m/K7 Ac
724.369069*	z_5	0.81	K16 3m/K7 Ac

887.431657*	z_6	-0.17	K16 3m/K7 Ac
903.450559*	y_6	0.03	K16 3m/K7 Ac
1043.53325*	z_7	0.32	K16 3m/K7 Ac
522.270083*	z_7^{2+}	-0.03	K16 3m/K7 Ac
703.372952*	z_{10}^{2+}	-0.66	K16 3m/K7 Ac
525.965412	z_{11}^{3+}	0.13	K16 3m
558.988583	z_{12}^{3+}	0.78	K16 3m
577.996078	z_{13}^{3+}	1.34	K16 3m
597.002734	z_{14}^{3+}	0.47	K16 3m
630.684924	z_{15}^{3+}	-0.14	K16 3m
710.39298	z_{18}^{3+}	-1.46	K16 3m
533.04669	z_{18}^{4+}	-1.20	K16 3m
834.472643	$z_{21}^{3+\bullet}$	-0.01	K16 3m
625.854063	z_{21}^{4+}	-0.47	K16 3m
643.613494	z_{22}^{4+}	-0.21	K16 3m
661.373351	z_{23}^{4+}	0.67	K16 3m
693.396715	z_{24}^{4+}	0.09	K16 3m
554.92081	z_{24}^{5+}	3.66	K16 3m
482.11074	y_{25}^{6+}	-1.08	K16 3m
575.125734	z_{25}^{5+}	-4.48	K16 3m
786.49366*	c_7	-1.00	K7 Ac
873.528202	c_8	1.98	K7 Ac
521.312289	c_{10}^{2+}	0.91	K7 Ac
556.829235	c_{11}^{2+}	-2.04	K7 Ac
607.355057	c_{12}^{2+}	1.39	K7 Ac
635.865738	c_{13}^{2+}	1.25	K7 Ac
664.376031	c_{14}^{2+}	0.54	K7 Ac
713.910047	c_{15}^{2+}	0.23	K7 Ac
777.957533	c_{16}^{2+}	0.22	K7 Ac
518.974127	c_{16}^{3+}	0.24	K7 Ac
959.058604	c_{19}^{2+}	-2.17	K7 Ac
639.710411	c_{19}^{3+}	1.35	K7 Ac
480.040265	c_{19}^{4+}	13.09	K7 Ac
519.060642	c_{20}^{4+}	2.67	K7 Ac
559.825756	c_{21}^{4+}	1.19	K7 Ac
622.863453	$c_{23}^{4+\bullet}$	2.97	K7 Ac
637.115589	$c_{24}^{4+\bullet}$	-2.16	K7 Ac
651.62492	c_{25}^{4+}	0.97	K7 Ac
544.97191	z_{12}^{3+}	-1.07	K7 Ac
550.313714	y_{12}^{3+}	2.99	K7 Ac
563.978366	z_{13}^{3+}	-2.28	K7 Ac
582.987558	z_{14}^{3+}	1.29	K7 Ac
616.666305	z_{15}^{3+}	-4.96	K7 Ac
508.780281	y_{17}^{4+}	-1.40	K7 Ac

522.534946	z_{18}^{4+}	-1.23	K7 Ac
587.069597	$z_{20}^{4+•}$	-3.98	K7 Ac
834.460358	$z_{21}^{3+•}$	-0.20	K7 Ac
625.846914	z_{21}^{4+}	2.65	K7 Ac
643.604573	z_{22}^{4+}	0.06	K7 Ac
661.361942	z_{23}^{4+}	-2.83	K7 Ac
665.371063	y_{23}^{4+}	3.86	K7 Ac
693.388356	z_{24}^{4+}	1.16	K7 Ac
554.913939	z_{24}^{5+}	4.40	K7 Ac
462.593144	z_{24}^{6+}	-2.13	K7 Ac
575.320182	$z_{25}^{5+•}$	-4.20	K7 Ac
482.1062	y_{25}^{6+}	2.08	K7 Ac

* Peak used for internal calibration

Average of absolute value of mass accuracy: 1.4 ppm

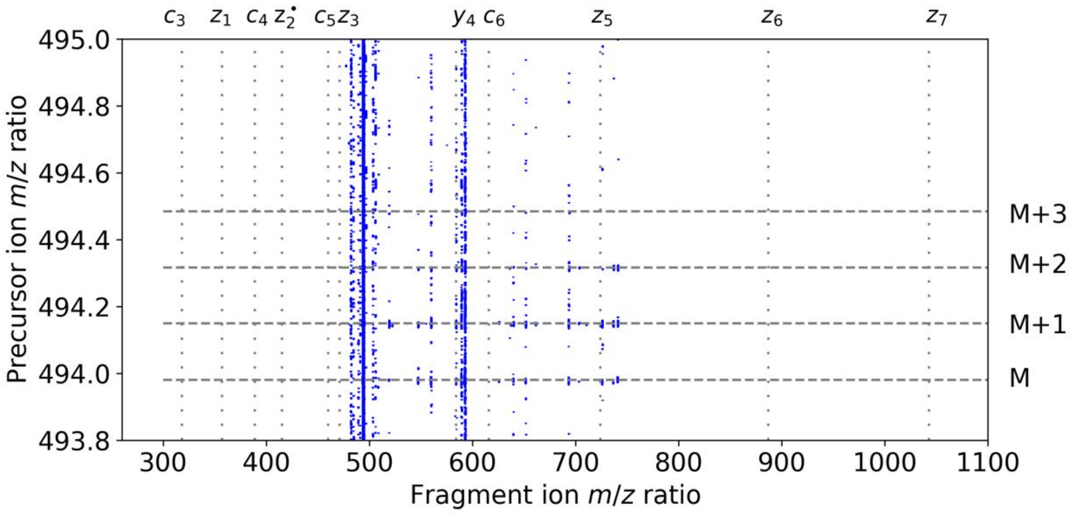

Figure S5. 2D mass spectrum of the K7 Ac/K16 3m histone peptide mixture with a frequency range of 1 kHz.

Table S4. Peak assignment of the 2D mass spectrum with f_N = 1 kHz.

***m/z* ratio**	**Assignment**	**Mass accuracy (ppm)**	**Precursor**
318.213469*	c_3	-0.35	K16 3m/K7 Ac
389.250928*	c_4	0.60	K16 3m/K7 Ac
460.287842*	c_5	0.07	K16 3m/K7 Ac
616.388591*	c_6	-0.53	K16 3m/K7 Ac
744.485492	c_7	2.16	K16 3m
831.517972	c_8	2.48	K16 3m
416.261722	c_8^{2+}	0.31	K16 3m
500.306353	c_{10}^{2+}	-0.36	K16 3m
535.824621	c_{11}^{2+}	-0.87	K16 3m
586.347921	c_{12}^{2+}	-1.72	K16 3m
614.860108	c_{13}^{2+}	0.73	K16 3m
643.370246	c_{14}^{2+}	-0.23	K16 3m
518.985713	c_{16}^{3+}	-0.80	K16 3m
639.721187	c_{19}^{3+}	-0.76	K16 3m
480.043353	c_{19}^{4+}	0.58	K16 3m
519.069105	c_{20}^{4+}	1.45	K16 3m
746.10762	c_{21}^{3+}	-2.95	K16 3m
559.834036	c_{21}^{4+}	-0.27	K16 3m
622.870381	$c_{23}^{4+\bullet}$	-0.51	K16 3m
637.127343	$c_{24}^{4+\bullet}$	2.01	K16 3m
651.633152	c_{25}^{4+}	-0.36	K16 3m
415.200502	$z_2^{\bullet}$	-1.12	K16 3m/K7 Ac
724.36865*	z_5	0.23	K16 3m/K7 Ac
887.433636	z_6	2.06	K16 3m/K7 Ac
903.453082	y_6	2.82	K16 3m/K7 Ac
1043.53288*	z_7	-0.04	K16 3m/K7 Ac

522.269432	z_7^{2+}	-1.28	K16 3m/K7 Ac
590.800421	z_8^{2+}	1.47	K16 3m/K7 Ac
647.33646	y_9^{2+}	1.79	K16 3m/K7 Ac
703.373009*	z_{10}^{2+}	-0.58	K16 3m/K7 Ac
525.964334	z_{11}^{3+}	-1.92	K16 3m
559.32081	$z_{12}^{3+\bullet}$	-5.86	K16 3m
577.99484	z_{13}^{3+}	-0.80	K16 3m
597.002618	z_{14}^{3+}	0.27	K16 3m
631.022814	$z_{15}^{3+\bullet}$	2.94	K16 3m
710.396664	z_{18}^{3+}	3.73	K16 3m
533.048409	z_{18}^{4+}	2.03	K16 3m
587.08384	$z_{20}^{4+\bullet}$	4.78	K16 3m
626.104107	$z_{21}^{4+\bullet}$	-3.52	K16 3m
643.608328	z_{22}^{4+}	-8.24	K16 3m
881.494547	z_{23}^{3+}	-0.27	K16 3m
661.373196	z_{23}^{4+}	0.43	K16 3m
693.396201	z_{24}^{4+}	-0.65	K16 3m
555.11888	$z_{24}^{5+\bullet}$	-2.63	K16 3m
482.112192	y_{25}^{6+}	1.93	K16 3m
575.327277	$z_{25}^{5+\bullet}$	-4.52	K16 3m
786.495105*	c_7	0.84	K7 Ac
873.52627*	c_8	-0.24	K7 Ac
521.310506	c_{10}^{2+}	-2.51	K7 Ac
548.317698	b_{11}^{2+}	1.10	K7 Ac
556.830659	c_{11}^{2+}	0.52	K7 Ac
607.353807	c_{12}^{2+}	-0.67	K7 Ac
635.86473	c_{13}^{2+}	-0.33	K7 Ac
664.37559	c_{14}^{2+}	-0.13	K7 Ac
713.911259	c_{15}^{2+}	1.93	K7 Ac
777.95834	c_{16}^{2+}	1.26	K7 Ac
518.973634	c_{16}^{3+}	-0.71	K7 Ac
959.061131	c_{19}^{2+}	0.47	K7 Ac
639.709363	c_{19}^{3+}	-0.29	K7 Ac
480.038702	c_{19}^{4+}	9.84	K7 Ac
519.059123	c_{20}^{4+}	-0.26	K7 Ac
559.825846	c_{21}^{4+}	1.35	K7 Ac
623.11161	c_{23}^{4+}	-3.13	K7 Ac
637.369041	c_{24}^{4+}	0.18	K7 Ac
651.625026	c_{25}^{4+}	1.13	K7 Ac
517.287797	y_{11}^{3+}	-2.84	K7 Ac
550.312	y_{12}^{3+}	-0.13	K7 Ac
544.972229	z_{12}^{3+}	-0.49	K7 Ac
564.314751	$z_{13}^{3+\bullet}$	-1.49	K7 Ac
874.476636	$z_{14}^{2+\bullet}$	-4.40	K7 Ac

582.987117	z_{14}^{3+}	0.53	K7 Ac
616.670394	z_{15}^{3+}	1.67	K7 Ac
508.783813	y_{17}^{4+}	5.54	K7 Ac
696.715672	$z_{18}^{3+\bullet}$	1.96	K7 Ac
522.534007	z_{18}^{4+}	-3.03	K7 Ac
544.546038	$z_{19}^{4+\bullet}$	0.89	K7 Ac
587.071732	$z_{20}^{4+\bullet}$	-0.35	K7 Ac
626.097067	$z_{21}^{4+\bullet}$	-0.23	K7 Ac
643.603087	z_{22}^{4+}	-2.25	K7 Ac
661.362847	z_{23}^{4+}	-1.46	K7 Ac
693.388455	z_{24}^{4+}	1.30	K7 Ac
554.909295	z_{24}^{5+}	-3.97	K7 Ac
575.322216	$z_{25}^{5+\bullet}$	-0.67	K7 Ac
482.105973	y_{25}^{6+}	1.61	K7 Ac

* Peak used for internal calibration
Average of absolute value of mass accuracy: 1.6 ppm

Table S5. Theoretical mass and relative abundance of the M and M+1 isotopes of the K7 Ac histone peptide.

mass (Da)	Relative Abundance (a.u.)	Chemical composition
2958.63246	0.17945051	$^{12}C_{129}{}^{1}H_{217}{}^{14}N_{44}{}^{16}O_{34}{}^{32}S$
2959.63581	0.2522669	$^{12}C_{128}{}^{13}C^{1}H_{217}{}^{14}N_{44}{}^{16}O_{34}{}^{32}S$
2959.63184	0.0014206	$^{12}C_{129}{}^{1}H_{217}{}^{14}N_{44}{}^{16}O_{34}{}^{33}S$
2959.63667	0.00233026	$^{12}C_{129}{}^{1}H_{216}{}^{2}H^{14}N_{44}{}^{16}O_{34}{}^{32}S$
2959.62949	0.0288617	$^{12}C_{129}{}^{1}H_{217}{}^{14}N_{43}{}^{15}N^{16}O_{34}{}^{32}S$
2959.63873	0.00450597	$^{12}C_{129}{}^{1}H_{217}{}^{14}N_{44}{}^{17}O_{34}{}^{32}S$

Table S6. Theoretical mass and relative abundance of the M and M+1 isotopes of the K16 3m histone peptide (calculated with FAST-MS, a software for top-down analysis that was developed at the University of Innsbruck (https://github.com/michael-palasser/FAST-MS).

mass (Da)	Relative Abundance (a.u.)	Chemical composition
2958.66884	0.17786582	$^{12}C_{130}{}^{1}H_{221}{}^{14}N_{44}{}^{16}O_{33}{}^{32}S$
2959.6722	0.25197748	$^{12}C_{129}{}^{13}C^{1}H_{221}{}^{14}N_{44}{}^{16}O_{33}{}^{32}S$
2959.66823	0.00140806	$^{12}C_{130}{}^{1}H_{221}{}^{14}N_{44}{}^{16}O_{33}{}^{33}S$
2959.67306	0.00224175	$^{12}C_{130}{}^{1}H_{220}{}^{2}H^{14}N_{44}{}^{16}O_{33}{}^{32}S$
2959.66588	0.02860683	$^{12}C_{130}{}^{1}H_{221}{}^{14}N_{43}{}^{15}N^{16}O_{33}{}^{32}S$
2959.67512	0.0045485	$^{12}C_{130}{}^{1}H_{221}{}^{14}N_{44}{}^{16}O_{32}{}^{17}O^{32}S$

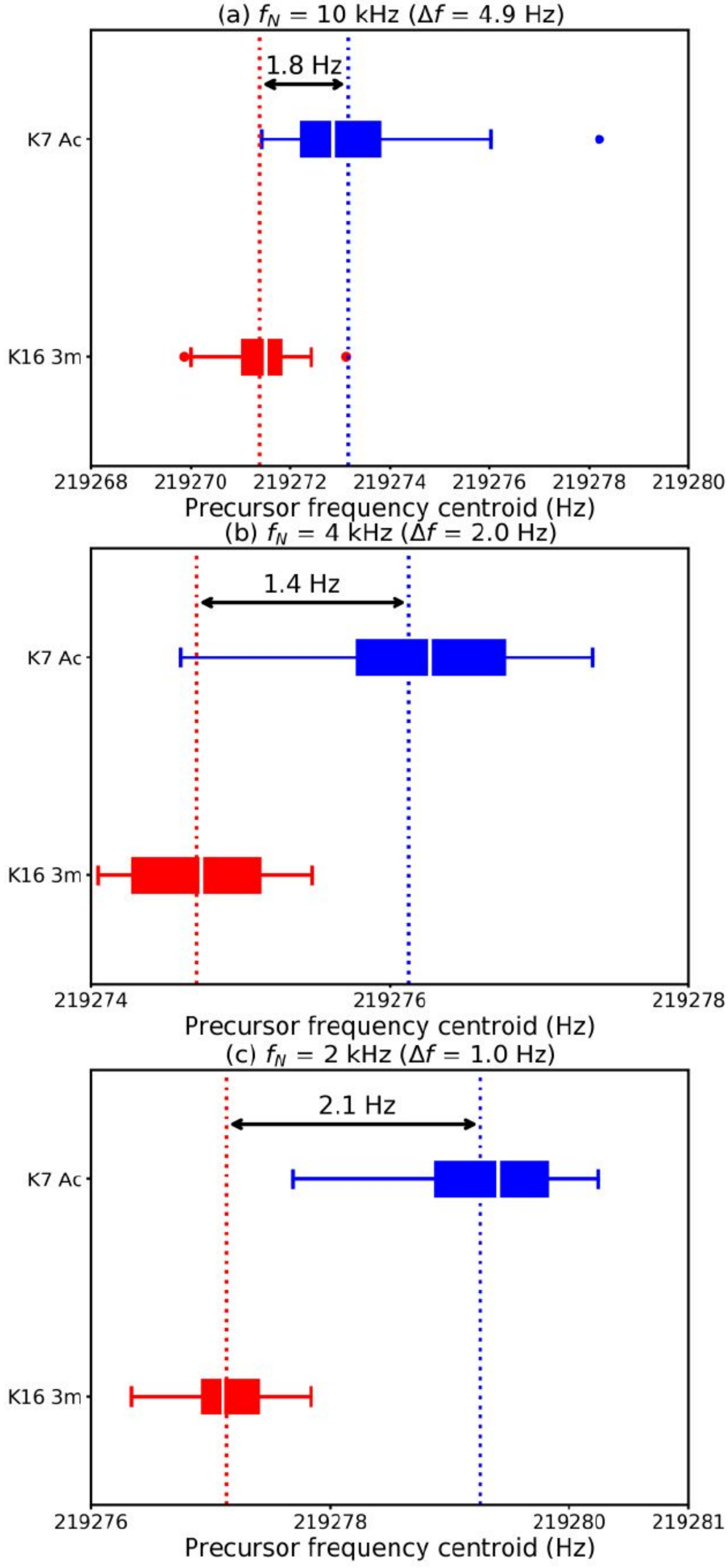
(a) f_N = 10 kHz (Δf = 4.9 Hz)
1.8 Hz
K7 Ac
K16 3m
219268
219270
219272
219274
219276
219278
219280
Precursor frequency centroid (Hz)
(b) f_N = 4 kHz (Δf = 2.0 Hz)
1.4 Hz
K7 Ac
K16 3m
219274
219276
219278
Precursor frequency centroid (Hz)
(c) f_N = 2 kHz (Δf = 1.0 Hz)
2.1 Hz
K7 Ac
K16 3m
219276
219278
219280
219281
Precursor frequency centroid (Hz)

Figure S6. Distribution of frequencies of fragment ion peaks (vertical precursor ion dimension) after Lorentzian fit for fragment ions $c_{16\text{-}25}$ and $y/z_{19\text{-}25}$ of the K16 3m (red) and the K7 Ac (blue) histone peptides (M isotopic peaks only). The vertical dotted lines indicate the average frequency measured for the fragments of K16 3m (red) and K7 Ac (blue). These measurements were performed on the 2D mass spectra acquired with (a) 10 kHz frequency range, (b) 4 kHz frequency range, and (c) 2 kHz frequency range.

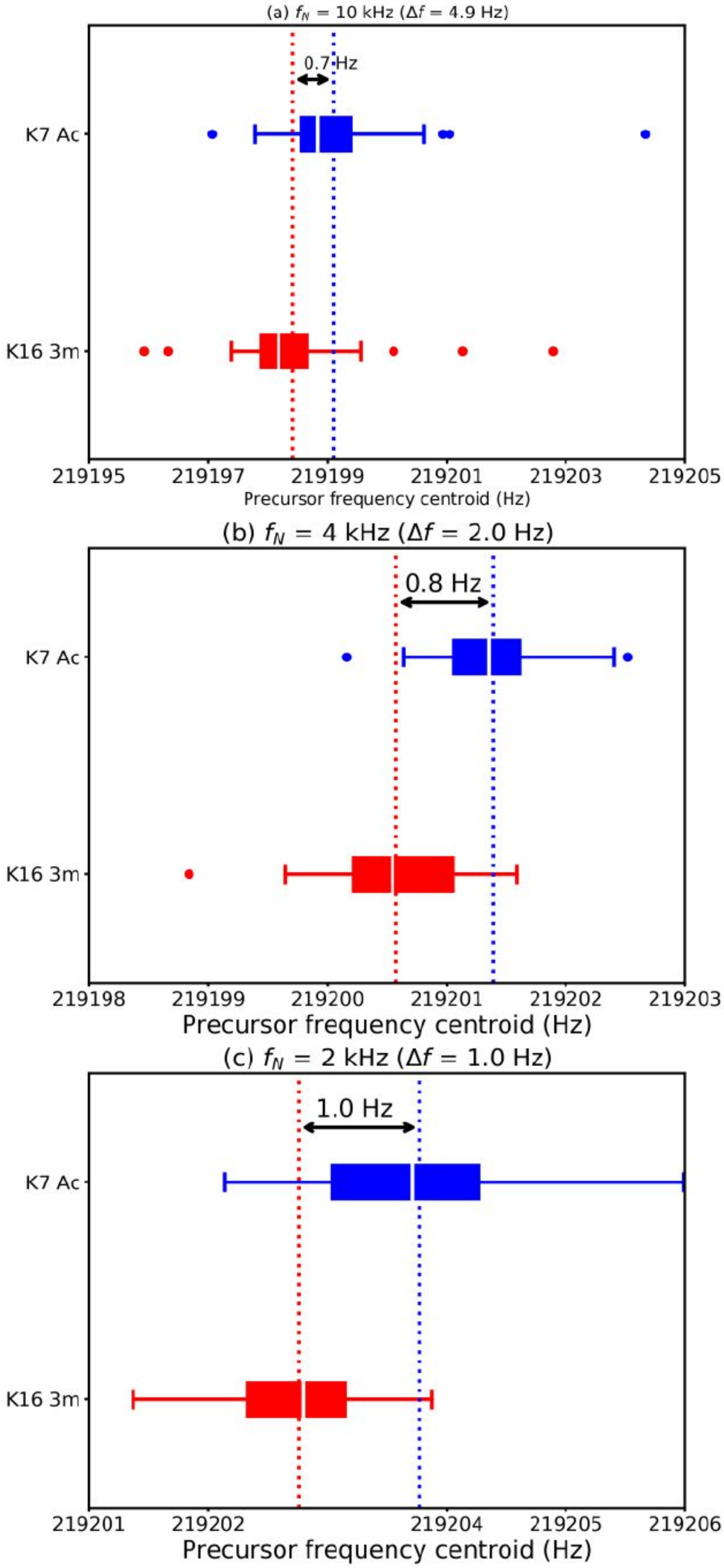

(a) f_N = 10 kHz (Δf = 4.9 Hz)
0.7 Hz
K7 Ac
K16 3m
219195
219197
219199
219201
219203
219205
Precursor frequency centroid (Hz)
(b) f_N = 4 kHz (Δf = 2.0 Hz)
0.8 Hz
K7 Ac
K16 3m
219198
219199
219200
219201
219202
219203
Precursor frequency centroid (Hz)
(c) f_N = 2 kHz (Δf = 1.0 Hz)
1.0 Hz
K7 Ac
K16 3m
219201
219202
219204
219205
219206
Precursor frequency centroid (Hz)

Figure S7. Distribution of frequencies of fragment ion peaks (vertical precursor ion dimension) after Lorentzian fit for fragment ions $c_{7\text{-}15}$ and $y/z_{11\text{-}18}$ of the K16 3m (red) and the K7 Ac (blue) histone peptides (M+1 isotopic peaks only). The vertical dotted lines indicate the average frequency measured for the fragments of K16 3m (red) and K7 Ac (blue). These measurements were performed on the 2D mass spectra acquired with (a) 10 kHz frequency range, (b) 4 kHz frequency range, and (c) 2 kHz frequency range.
